# Nanoparticle-Enabled Biomaterials for Controlled Drug Delivery in Implantable and Wearable Devices

**DOI:** 10.3390/ijms27167265

**Published:** 2026-08-14

**Authors:** Zahrah Asiri, Abeer Mobarki, Sahar. S Alghamdi, Abdulaziz A. Almoutairi, Fatimah Alsalman, Rawan Fitaihi, Njoud Altuwaijri, Arwa Alsubait, Yahya F. Jamous

**Affiliations:** 1 Department of Pharmaceutics, College of Pharmacy, King Khalid University, Abha 62529, Saudi Arabia; z.asiri@kku.edu.sa; 2 Department of Maternal and Child Health Nursing, College of Nursing, King Saud University, Riyadh 12372, Saudi Arabia; 3 College of Pharmacy, King Saud bin Abdulaziz University for Health Sciences, Riyadh 11481, Saudi Arabia; 4 Ministry of National Guard Health Affairs (MNGHA), Riyadh 11426, Saudi Arabia; 5 Medical Research Core Facility and Platforms Department, King Abdullah International Medical Research Center (KAIMRC), Riyadh 11481, Saudi Arabia; 6 Nursing Professional Development Department, Dammam Medical Complex, Dammam 32245, Saudi Arabia; 7 Department of Pharmaceutics, College of Pharmacy, King Saud University, Riyadh 11451, Saudi Arabia; 8 Department of Clinical Laboratory Sciences, College of Applied Medical Sciences, King Saud bin Abdulaziz University for Health Sciences, Riyadh 11481, Saudi Arabia; 9 Wellness and Preventive Medicine Institute, Health Sector, King Abdulaziz City for Science and Technology (KACST), Riyadh 11442, Saudi Arabia

**Keywords:** nanoparticle-based biomaterials, biomaterials, implantable devices, wearable devices, controlled drug delivery, stimuli-responsive release, microneedles, drug-eluting implants, closed-loop systems

## Abstract

Conventional oral and injectable drug administration still struggles with unstable plasma levels, weak targeting, and considerable systemic toxicity, problems that become especially acute in chronic disease management. Implantable and wearable biomedical devices offer one path around these limits, yet device-only platforms continue to fall short on drug loading, release control, and protection of fragile therapeutics. Integrating nanoparticle-based biomaterials into such devices has therefore moved from a research curiosity to a serious clinical strategy. As a result, understanding the design principles, translational challenges, and clinical potential of these hybrid platforms has become increasingly important. This review provides a comprehensive assessment of four major nanoparticle families—polymeric carriers (PLGA, chitosan, and micelles), lipid-based vehicles (liposomes, SLNs, and NLCs), inorganic systems (gold, mesoporous silica, iron oxide, and calcium phosphate), and hybrid composites—focusing on how their physicochemical properties govern drug encapsulation, release behavior, and tissue compatibility. These classes are then linked to specific implantable formats such as drug-eluting stents, nano-enabled scaffolds, and reservoir depots, and to wearable formats including transdermal patches, microneedle arrays, biosensor-coupled patches, and patient-actuated devices. A dedicated section addresses stimuli-responsive release driven by pH, enzymes, temperature, and electrical or magnetic fields, alongside closed-loop platforms that pair real-time biosensing with on-demand dosing. Surface engineering strategies, ligand targeting, antifouling coatings, antimicrobial layers, and immune-modulating chemistries are also discussed, together with the central translational hurdles: long-term stability, foreign body response, scale-up, sterilization, and regulatory classification of combination products. Finally, the review outlines near-term directions, including AI-driven dosing, 4D bioprinting, biomimetic nanocarriers, gene therapy delivery, and bioresorbable electronics, that together suggest where these hybrid platforms are likely to mature next.

## 1. Introduction

Nanoparticle-enabled biomaterials integrated with implantable and wearable devices represent an increasingly important approach to achieving sustained, localized, and patient-adapted drug delivery while reducing the limitations associated with conventional systemic administration [1,2,3,4,5,6,7]. The distinguishing feature of the present review is its integrated evaluation of nanoparticle composition, device architecture, release behavior, biointerface engineering, long-term safety, manufacturing feasibility, and regulatory translation. Accordingly, this review aims to critically compare the principal nanoparticle-based biomaterial classes, examine their incorporation into implantable and wearable platforms, evaluate their advantages and limitations, and identify the factors most likely to influence their future clinical adoption.

### 1.1. Clinical Need for Advanced Drug Delivery Systems

Effective drug delivery remains one of the enduring challenges of modern therapeutics, particularly for diseases that demand sustained, long-term treatment. Although oral and injectable routes still dominate clinical practice, they frequently produce pronounced fluctuations in plasma drug concentrations, offer limited capacity for site-specific targeting, and carry a substantial risk of systemic toxicity [1,2,3,4]. Such shortcomings erode therapeutic efficacy and undermine patient adherence, especially when treatment requires frequent dosing over prolonged periods. As the global burden of chronic disease continues to grow, too does the need for delivery strategies that produce sustained, precise, and predictable therapeutic effects. This need is most acute in conditions such as diabetes, cancer, cardiovascular disease, and neurological disorders, where drug concentrations must be held within a narrow therapeutic window: systemic overexposure precipitates toxicity, whereas underexposure compromises efficacy. Delivery platforms that combine controlled release with minimal systemic exposure have consequently become a central objective of contemporary biomedical and pharmaceutical research [1,2,3,4].

### 1.2. Importance of Implantable and Wearable Biomedical Devices

**Figure 1:**
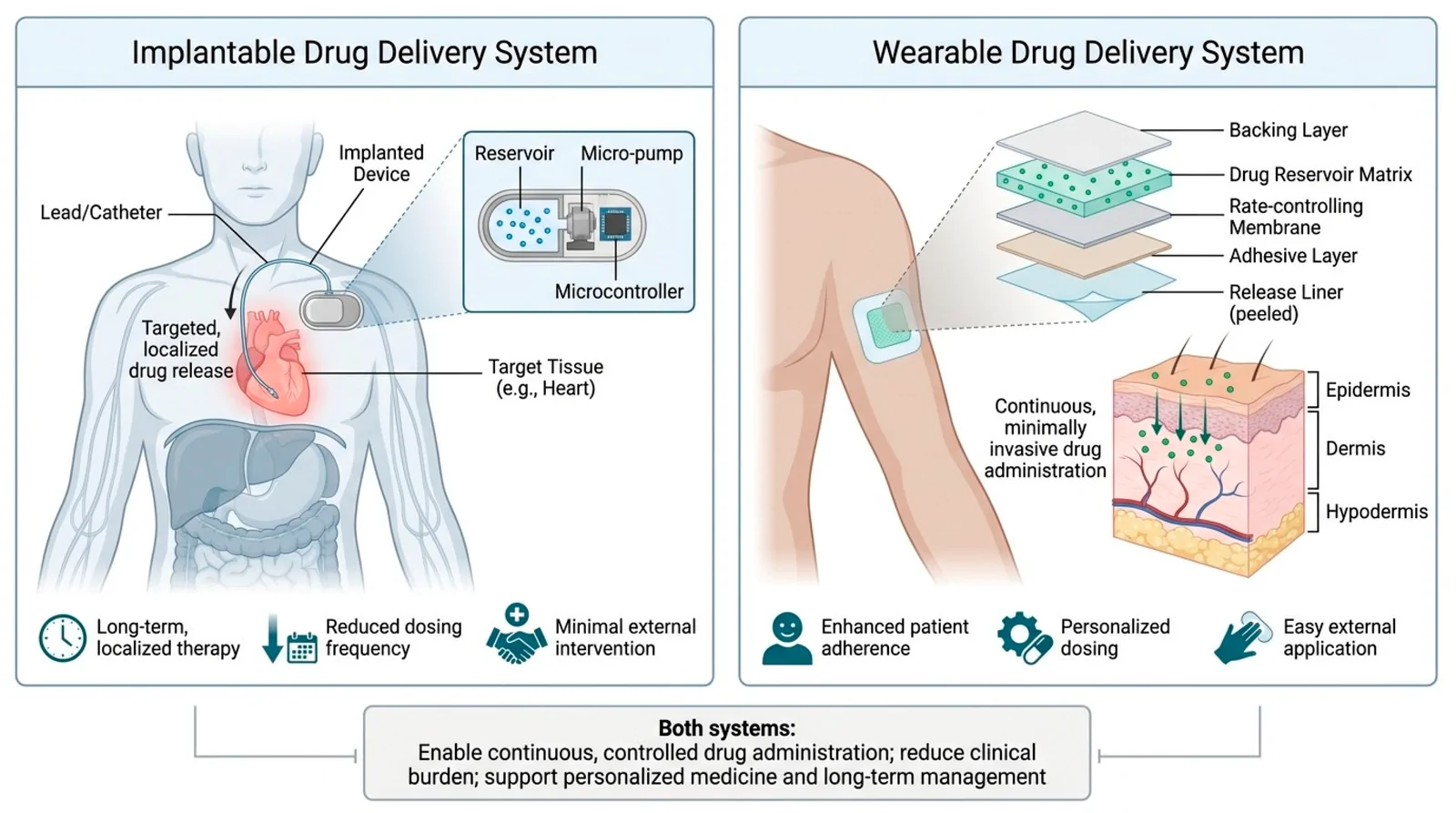
Implantable and wearable biomedical devices for controlled drug delivery. Implantable platforms enable long-term localized therapy, while wearable transdermal systems facilitate minimally invasive and sustained drug administration.

Implantable and wearable biomedical devices have emerged as compelling responses to these limitations. Wearable systems such as transdermal patches and microneedle-based platforms permit minimally invasive, continuous drug administration (Figure 1), easing patient burden, improving adherence, and reducing reliance on frequent injections or complex dosing regimens; in doing so, they extend disease management well beyond the clinical setting [5,6,7]. Implantable devices offer a complementary advantage, delivering therapeutics directly to the disease site over extended intervals. Advances in miniaturization, materials engineering, and device control now allow such systems to release drugs over weeks to months with minimal external intervention. Taken together, both platforms mark meaningful progress toward patient-specific therapy and align with the broader goals of personalized medicine and continuous, integrated care [5,6,7].

### 1.3. Limitations of Conventional and Device-Only Drug Delivery

**Figure 2:**
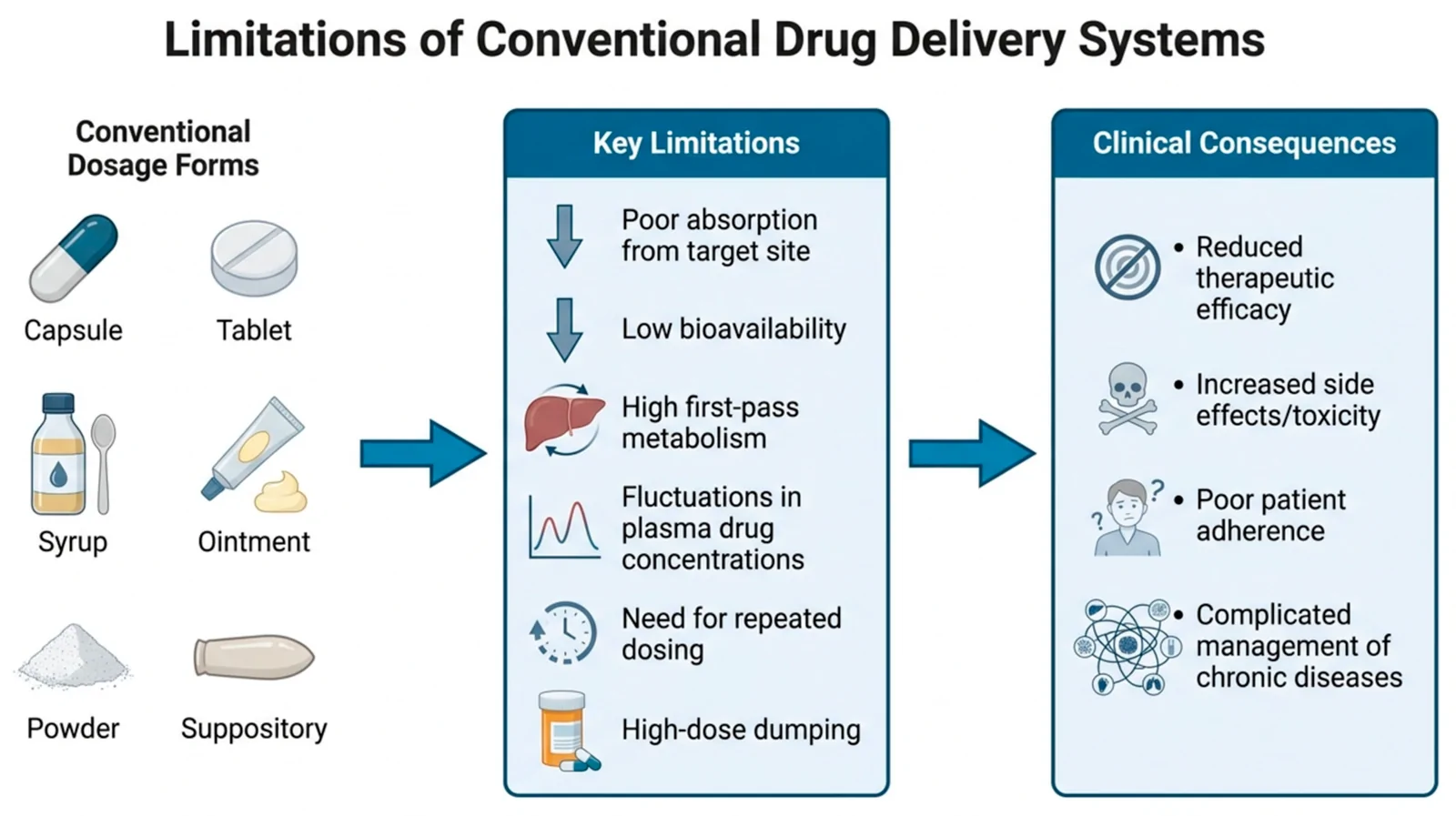
Limitations of conventional drug delivery systems. Conventional dosage forms are often associated with poor bioavailability, first-pass metabolism, fluctuating plasma drug concentrations, repeated dosing requirements, and limited site-specific targeting.

Despite this progress, delivery systems that rely on device architecture alone remain limited when they are not paired with advanced materials or carrier technologies. Their principal constraints are a restricted drug-loading capacity, imprecise control over release kinetics, and an inability to stabilize sensitive therapeutic agents such as proteins and nucleic acids. Compounding these issues, the foreign body response provoked by implanted materials can drive fibrotic encapsulation that progressively degrades long-term performance. These constraints call for strategies that simultaneously stabilize the drug, govern its release, and temper adverse biological responses; neglecting them shortens the useful life of device-based therapy and reinforces the case for integrating advanced materials and carrier systems into device design [5,6,8,9] (Figure 2).

### 1.4. Role of Nanotechnology in Advancing Smart Drug Delivery Systems

Nanotechnology has proved pivotal in addressing these challenges, furnishing nanoscale carriers that enhance drug stability, targeting, and controlled release. Nanoparticles can encapsulate therapeutic agents and shield them from premature degradation, and their release can be tuned by deliberate modulation of material properties. Their small dimensions and adaptable surface chemistry mediate rich interactions with biological systems, from cellular uptake to tissue integration. Most importantly, nanocarriers can be engineered to respond to endogenous physiological cues or to external stimuli such as pH, enzymatic activity, temperature, or electrical signals, so that drug release adjusts dynamically as physiological conditions change, marking a decisive step toward personalized and adaptive therapy [3,10,11].

### 1.5. Advantages of Nanotechnology-Based Biomaterials

Although nanoparticles offer distinctive physicochemical properties, their clinical utility improves markedly when they are paired with biocompatible biomaterials that enhance stability, safety, and tissue integration. Nanotechnology-based biomaterials unite the functional attributes of nanoparticles with the structural and biological features of biomaterial matrices, yielding hybrid systems that support safe drug release, attenuate immune responses, and promote tissue compatibility. Biodegradable polymers and hydrogels, for example, can be engineered to regulate nanoparticle accumulation, degradation, and release kinetics. In this way, nanotechnology-based biomaterials bridge nanoscale functionality and in vivo performance by effectively interfacing nanoparticles with the biological environment [2,6,12].

### 1.6. Integration of Nanoparticle-Based Biomaterials with Implantable and Wearable Devices

Integrating nanoparticle-based biomaterials with implantable and wearable devices offers a particularly promising route to precise, controlled drug delivery. In wearable systems, nanoparticles embedded within polymeric matrices or microneedles enable controlled, sustained transdermal release; in implantable devices, nanoparticle-loaded biomaterials localize therapy at the target site while limiting systemic exposure. By combining the strengths of devices, nanotechnology, and biomaterials, these hybrid platforms yield multifunctional systems capable of long-term, responsive, and targeted therapy, representing a pragmatic advance toward the next generation of smart drug delivery [2,3,5,13]. Building on this rationale, the present review examines the major classes of nanoparticle-based biomaterials and their defining properties, their integration into implantable and wearable formats, the mechanisms that govern controlled and stimuli-responsive release, and the surface-engineering, translational, and regulatory considerations that will ultimately shape their clinical adoption.

## 2. Nanoparticle-Based Biomaterials: Classes and Properties

The diversity of nanoparticle-based biomaterials available for controlled drug delivery in implantable and wearable devices reflects decades of innovation at the intersection of polymer chemistry, lipid science, inorganic materials engineering, and hybrid nanoscience. Each class of nanoparticle offers distinct physicochemical properties that govern drug encapsulation efficiency, release kinetics, biocompatibility, and suitability for integration into biomedical devices. Understanding these material classes and their inherent properties is foundational to designing effective drug delivery platforms tailored to specific clinical applications.

### 2.1. Polymer-Based Nanoparticles

Polymer-based nanoparticles represent one of the most extensively investigated classes of nanocarriers because of their structural versatility, tunable size, adjustable surface chemistry, and programmable degradation behavior. Both natural and synthetic polymers have been used to fabricate nanoparticulate drug carriers. Synthetic biodegradable polymers, including poly(lactic-co-glycolic acid) (PLGA), polylactic acid (PLA), polycaprolactone (PCL), and polyethylene glycol (PEG)-based systems, are widely investigated because of their well-characterized physicochemical properties and established use in biomedical applications [14,15,16]. Natural polymers such as chitosan, alginate, gelatin, hyaluronic acid, and silk fibroin provide additional advantages, including biocompatibility, mucoadhesion, ionic crosslinking capacity, and intrinsic biological activity [17]. Polymeric micelles extend this class through core–shell architectures that permit the incorporation of therapeutics with different physicochemical characteristics. Overall, polymer composition, molecular weight, crystallinity, surface charge, and degradation profile determine drug compatibility, nanoparticle stability, and suitability for incorporation into implantable and wearable biomaterial matrices. Detailed passive and stimuli-responsive drug-release mechanisms are discussed separately in Section 5.

Recent work on rational polymeric micelle design has demonstrated the ability to tune targeting specificity and pharmacokinetic profiles through systematic variation in polymer composition and surface ligand density [18]. The ability to functionalize polymer nanoparticles with targeting ligands, PEGylation layers, and stimuli-responsive moieties makes them exceptionally adaptable to the complex demands of wearable and implantable biomedical devices.

### 2.2. Lipid-Based Nanoparticles

Lipid-based nanoparticles, including liposomes, solid lipid nanoparticles (SLNs), nanostructured lipid carriers (NLCs), and lipid-polymer hybrid systems, constitute a clinically advanced category of drug delivery vehicles with favorable biocompatibility profiles arising from their structural similarity to biological membranes [19]. Liposomes are spherical vesicles composed of one or more phospholipid bilayers enclosing an aqueous core, enabling co-encapsulation of both hydrophilic agents in the core and lipophilic drugs within the bilayer. This amphiphilic architecture affords remarkable flexibility in drug loading and release modulation. The surface of liposomes can be readily modified with PEG to extend circulation half-life and reduce protein adsorption—a critical advantage in implantable contexts where foreign body response must be minimized. Contemporary liposomal research has focused on stimuli-responsive formulations, including thermosensitive liposomes capable of releasing more than 95% of their payload within seconds of reaching target temperatures, as well as pH-responsive systems tailored for tumor microenvironment-triggered release [20].

Solid lipid nanoparticles replace liquid lipid cores with solid lipid matrices, offering improved physical stability and more controlled drug release compared to emulsion systems. Nanostructured lipid carriers represent a further refinement, incorporating both solid and liquid lipids to create a less ordered matrix that improves drug-loading capacity and prevents drug expulsion during storage. A comprehensive analysis of lipid nanoparticle subtypes has confirmed that SLNs and NLCs offer superior encapsulation of poorly soluble drugs due to their high lipid core packing density, while also presenting scalable manufacturing options compatible with regulatory requirements [21]. The biocompatibility of lipid-based nanoparticles, combined with their capacity for formulation using FDA-approved excipients, positions them as highly translatable platforms for integration into wearable drug delivery patches and implantable reservoir systems.

### 2.3. Inorganic Nanoparticles

Inorganic nanoparticles, encompassing gold nanoparticles (AuNPs), mesoporous silica nanoparticles (MSNs), iron oxide nanoparticles, and calcium phosphate-based nanomaterials, offer unique physicochemical characteristics that complement organic counterparts and enable functionalities not achievable with polymer or lipid platforms alone [22]. Gold nanoparticles exhibit exceptional surface plasmon resonance properties that enable photo-triggered drug release upon near-infrared (NIR) irradiation, an attribute of considerable interest in on-demand delivery from implanted devices. Their well-defined thiol-gold surface chemistry allows precise conjugation of therapeutic molecules, antibodies, and targeting ligands.

Mesoporous silica nanoparticles are distinguished by their high surface area, tunable pore size, and large pore volume, enabling remarkably high drug loading capacities for diverse therapeutics. An extensive review of MSN biomedical applications published in 2023 highlighted that the porous architecture can be functionalized with pH-responsive gatekeepers, thermosensitive polymers, or magnetic elements to achieve stimuli-triggered release, and that MSN platforms are actively entering clinical trial phases for drug delivery and theranostic applications [23]. Iron oxide nanoparticles (IONPs), particularly superparamagnetic variants (SPIONs), are of dual interest as both drug carriers and diagnostic contrast agents—their zero coercivity, fast magnetic field response, and loss of magnetization upon field removal make them uniquely suitable for implantable systems requiring magnetically guided, on-demand drug release or combined hyperthermia-chemotherapy protocols [24]. Calcium phosphate nanoparticles, given their compositional resemblance to bone mineral, are particularly relevant for orthopedic implant applications, offering osteoconductive properties alongside drug delivery capability.

### 2.4. Hybrid and Composite Biomaterials

Recognizing that no single material class fulfills all requirements for advanced drug delivery devices, researchers have increasingly developed hybrid and composite nanoparticle systems that synergistically combine the advantageous properties of multiple material types [25]. Lipid-polymer hybrid nanoparticles (LPHNPs) integrate the structural stability and controlled release capacity of polymeric cores with the biocompatible lipid shell surface, achieving superior drug encapsulation efficiency alongside reduced immunogenicity. A comprehensive review of LPHNP design strategies, published in 2023, demonstrated that the outer lipid shell—particularly when incorporating DSPE-PEG—confers steric stabilization and prolongs systemic circulation, while the polymeric core enables high drug loading and sustained release profiles advantageous for implantable device applications [26].

**Figure 3:**
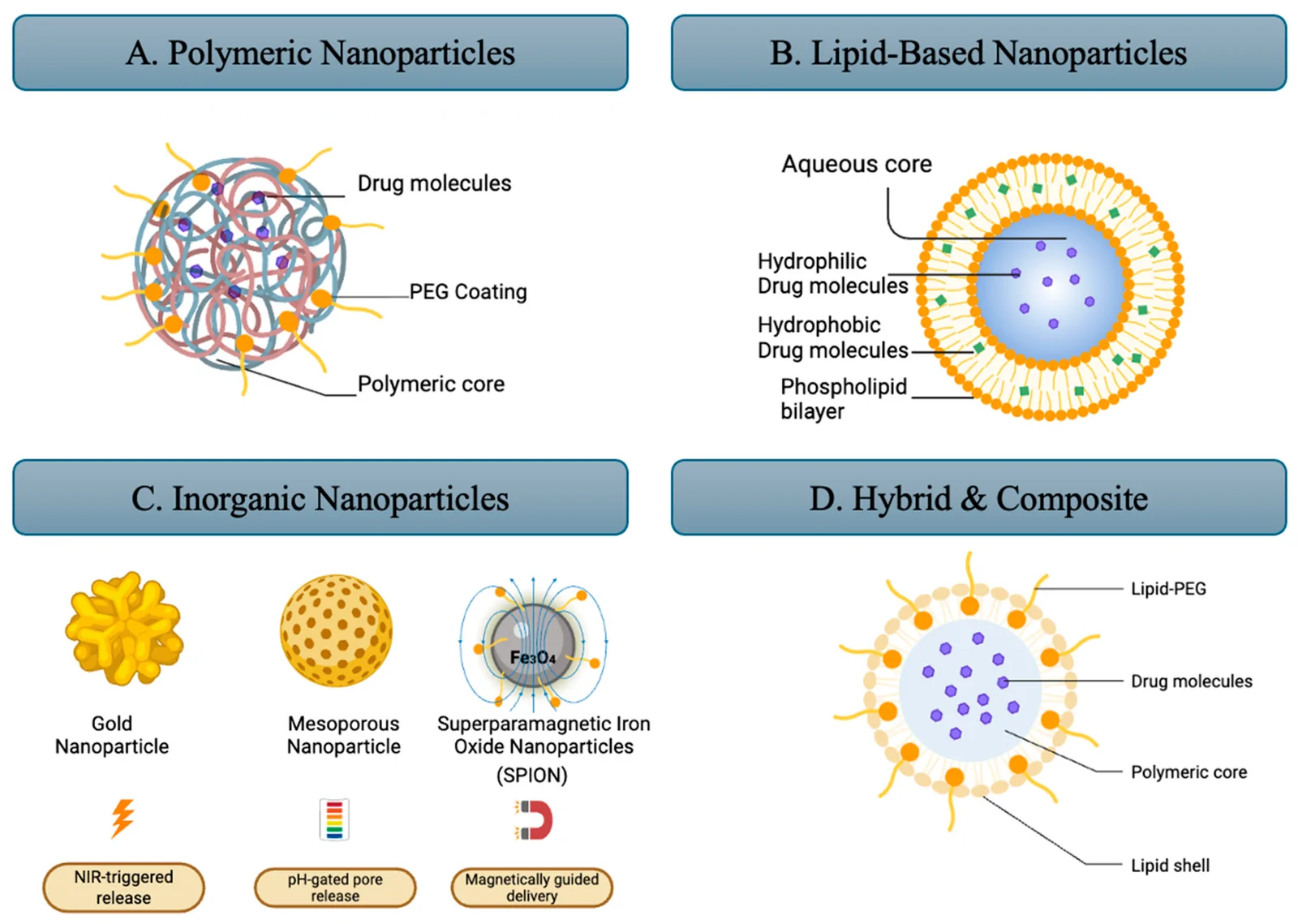
Classification of nanoparticle-based biomaterials. Representative polymeric, lipid-based, inorganic, and hybrid/composite nanoparticles are shown, highlighting their structural characteristics and functional roles in controlled drug delivery applications (Created with BioRender.com).

Hybrid magnetic systems combining SPIONs with lipid or polymeric matrices represent another critical dimension of hybrid design, particularly for implantable devices requiring remote programmability. Hybrid magnetic lipid-based nanoparticles exploiting SPIONs have been investigated for simultaneous chemotherapy, magnetic hyperthermia, and MRI-guided monitoring, demonstrating the theranostic potential of these multicomponent platforms [24]. Composite biomaterial scaffolds incorporating nanoparticles within hydrogel matrices, electrospun fiber networks, or ceramic substrates represent yet another design dimension highly relevant to implantable drug delivery systems. In these architectures, nanoparticles serve as discrete drug reservoirs distributed throughout a macroscopic scaffold, enabling sustained, localized release while the scaffold provides structural support for tissue integration. The combination of multiple nanoparticle types within a single composite platform can further enable multi-drug co-delivery with independently controlled release kinetics, addressing clinical needs for combinatorial therapeutic regimens [27] (Figure 3).

### 2.5. Biocompatibility and Biodegradability Considerations

Regardless of material class, biocompatibility and biodegradability are paramount considerations in the design of nanoparticle-based biomaterials for implantable and wearable applications, where prolonged tissue contact necessitates rigorous safety evaluation [28]. Biocompatibility encompasses cytotoxicity assessment as well as the evaluation of inflammatory responses, genotoxicity, complement activation, hemocompatibility, and protein corona formation, the last of which can dramatically alter nanoparticle pharmacokinetics and targeting efficiency in vivo. The protein corona, formed by plasma protein adsorption onto nanoparticle surfaces, confers a new biological identity on the nanocarrier that may trigger immune recognition, accelerate clearance, or—in certain formulations—be co-opted to enhance organ-specific targeting [29]. Surface charge, hydrophobicity, and curvature are among the principal physicochemical determinants of protein corona composition, and their systematic engineering is increasingly recognized as a critical tool for improving nanoparticle therapeutic performance.

Biodegradable materials must degrade into non-toxic byproducts at rates commensurate with drug release and tissue healing timelines, avoiding the accumulation of degradation intermediates that could provoke adverse tissue responses. Inorganic nanoparticles, which are generally non-biodegradable, require particular attention regarding long-term biopersistence and potential toxicity associated with accumulation in organs such as the liver and spleen. Biocompatibility testing of medical device materials is governed by the ISO 10993 series of standards, which mandates evaluation across endpoints including cytotoxicity, genotoxicity, immunotoxicity, hemocompatibility, and implantation response under ISO/TR 10993-22 for nanomaterials specifically [30]. Recent work has emphasized that standardized in vitro testing using physiologically relevant cell models, combined with rigorous in vivo pharmacokinetic and toxicological profiling, forms the essential experimental foundation for advancing nanoparticle-based biomaterials toward regulatory approval and clinical implementation. The principal characteristics of polymeric, lipid-based, inorganic, and hybrid nanoparticle-based biomaterials, together with their potential relevance to implantable and wearable drug delivery systems, are summarized in Table 1. Long-term safety considerations differ substantially across nanoparticle classes. Polymeric systems are generally designed to undergo hydrolytic or enzymatic degradation, but their safety depends on the rate of degradation, the local concentration of degradation products, and the capacity of surrounding tissues to clear these products. Lipid-based nanoparticles may exhibit more favorable biological compatibility because of their structural similarity to cellular membranes, although lipid oxidation, aggregation, and changes in surface composition can alter biological interactions over time. Inorganic nanoparticles require particular caution in long-term implantable applications because many are not readily biodegradable and may persist within tissues or accumulate in organs involved in mononuclear phagocyte clearance, including the liver and spleen. Surface charge, particle size, coating stability, and protein corona formation further influence biodistribution, immune recognition, cellular uptake, and clearance. Therefore, long-term evaluation should combine repeated-dose toxicity, biodistribution, organ accumulation, degradation-product analysis, immune-response profiling, and clearance studies under conditions that reflect the intended duration of device use [28,29,30].

### 2.6. Critical Comparison and Platform Selection

No single nanoparticle class is universally suitable for all implantable and wearable drug-delivery applications. Polymeric nanoparticles offer considerable flexibility in tuning degradation and sustained-release profiles, which may favor their use in biodegradable matrices and long-term delivery systems. Nevertheless, their performance can vary according to polymer composition, molecular characteristics, degradation behavior, and formulation conditions [14,15,16,17,18,28]. Lipid-based nanoparticles generally show favorable biocompatibility and can accommodate therapeutics with different physicochemical properties. However, lipid organization, storage-related instability, and potential drug expulsion may affect formulation performance over time [19,20,21]. Inorganic nanoparticles provide distinctive optical, magnetic, porous, or mineral-like properties that enable externally triggered release, imaging, and theranostic applications. Their use in long-term devices, however, requires careful assessment of biodegradability, tissue persistence, organ accumulation, and clearance [22,23,24,28,29,30]. Hybrid and composite systems can integrate complementary properties from more than one material class, thereby supporting multifunctional and multi-drug delivery. This added functionality is accompanied by greater compositional complexity and more demanding requirements for characterization, manufacturing reproducibility, and degradation assessment [24,25,26,27]. Accordingly, nanoparticle selection should be guided by the intended therapeutic application, payload characteristics, required release duration, device architecture, manufacturing feasibility, and long-term safety rather than by drug-loading capacity alone.

## 3. Implantable Drug Delivery Devices

Implantable drug delivery devices (IDDDs) play a critical role in achieving localized therapy, particularly for long-term therapy and in situations where systemic dosing produces unacceptable off-target toxicities. In the field of nanoparticle-based biomaterials, implantable systems are developed either by integrating drug-loaded nanoparticles into bulk matrices, such as hydrogels, biodegradable polymers, porous implants, or mineral scaffolds, or by applying nanoparticle-containing coatings to implantable devices to modulate release kinetics and improve tissue compatibility. These approaches facilitate prolonged drug concentrations at the target site, improve bioavailability, and reduce dosing frequency, thereby enhancing patient adherence and clinical outcomes. Depending on the nanoparticle type, surface chemistry, and carrier matrix, drug loading, diffusion pathways, degradation behavior, and ultimately the temporal release profile can be precisely tailored [31,32,33].

### 3.1. Drug-Eluting Implants

Drug-eluting implants are medical devices that slowly release drugs over a prolonged period. One of the most successful examples of clinical translation is the use of drug-eluting cardiovascular stents, which have become widely used to suppress neointimal hyperplasia and reduce restenosis. Primary evidence syntheses of alternative stent surface strategies and drug-eluting stents highlight the importance of the implant surface and coating design in balancing efficacy and safety in acute coronary syndrome (ACS) [34]. The application of nano-enabled biomaterials extends well beyond stents. For example, coatings containing nanoparticles and surface-bound nanocarriers can be used to sustain local concentrations near the implant and improve drug–tissue transfer at the implant interface. For instance, porous polyethylene implants modified with albumin nanocarriers represent a good model for surface nanoengineering to enable sustained localized factor release. This example demonstrates how nanocarriers and surface functionalization can convert a passive implant into an active drug-releasing biointerface [35].

The host foreign body response (FBR) is another important clinical factor that compromises the long-term performance of drug-eluting implants via chronic inflammation and fibrotic encapsulation. Biodegradable polymer implants have been used to modulate cellular responses via local delivery strategies. Intriguingly, localized delivery of pirfenidone through PLA implants is known to alter the foreign body response, strengthening the broader rationale that pharmacological modulation through an implanted device may improve integration and performance without causing systemic exposure [36]. To achieve predictable release from a drug-releasing implant, robust characterization of coating integrity and release behavior is crucial. Therefore, analytical frameworks for evaluating bioactive coatings and controlled release from implants are necessary for development and translation [37].

### 3.2. Nano-Enabled Scaffolds

Nano-enabled scaffolds provide structural functions, such as support, defect filling, and guidance for regeneration, while also enabling controlled drug release through incorporated nanoparticles. In key fields such as orthopedics and bone repair, a scaffold should impart mechanical, biological, and bioactive cues to prevent infection and inflammation. According to Burdușel and Andronescu [38] , lipid nanoparticles and liposomes are emerging as potential systems for bone-related diseases because they can encapsulate a variety of therapeutics. They can also be incorporated into scaffolds for controlled release. This approach enables sustained local drug concentrations within difficult-to-reach bone microenvironments. Scaffold composition and architecture are critical determinants of local drug delivery performance. For example, chitosan–hydrazone-modified calcium phosphate scaffolds have been characterized as drug-releasing constructs supporting localized and sustained release. This illustrates how scaffold chemistry and functional linkages can be tailored to modify release duration and local bioactivity [39]. Scaffolds reinforced with resveratrol-loaded solid lipid nanoparticles are another example where nanocarriers function as reservoirs within the scaffold network to allow the local delivery of bioactive compounds in a sustained manner while keeping the integrity and functionality of the scaffold intact [40].

Advanced manufacturing makes a stronger case for nano-enabled scaffolds. Three-dimensional bioprinting enables precise control over hydrogel organization and functionalization, thereby improving cell viability. A 3D bioprinted controlled-release scaffold made for the mesenchymal stem cell secretome shows that printing-enabled design can be combined with controlled delivery concepts that can support regenerative goals through sustained exposure to therapeutic factors [41]. Though not all such constructs would be “implantable” in every application, the platform logic is directly relevant: scaffolds can be implanted as engineered depots that couple tissue support with spatiotemporal therapeutic delivery.

### 3.3. Localized and Sustained Drug Release

The key benefit of implantable delivery systems is localized and sustained drug release. Mechanistically, sustained release is primarily achieved through hydrated polymer networks, matrix degradation/erosion or release from nanoparticle reservoirs within the carrier. Hydrogels are often highlighted as tunable matrices since their cross-linking density, swelling behavior, and degradability can be adjusted to fine-tune drug release over clinically relevant timeframes [42,43]. The introduction of nanoparticles or extracellular vesicles into a hydrogel alters binding interactions and diffusional constraints, providing an additional level of control over hydrogel behavior [43]. More recent studies extend beyond conventional polymer-based controlled release by introducing programmable delivery systems. For instance, DNA strand displacement circuits in hydrogels show how molecular programming can control release. The authors propose conceptual pathways for precision control in implantable matrices to allow on-demand dosing [44].

The target disease also influences the design requirements of sustained-release systems. Hydrogel-based formulations for localized analgesic delivery in chronic pain and postsurgical contexts are seen as routes to deliver drugs to sites of pain or inflammation while sustaining effect and limiting systemic side effects [45]. Hydrogel platforms are also described for osteoarthritis as local delivery systems with the capacity to provide sustained drug delivery within the joint, where such devices can be placed locally because of their anatomical suitability and prolonged exposure profiles [46]. Incorporating nanoparticles can enhance drug stability and facilitate the transfer of poorly soluble drugs. Likewise, matrix design determines how long the drug remains locally available [33,47].

### 3.4. Applications: Cancer, Orthopedics, Cardiovascular, Neurology

Nano-biomaterial depots and scaffolds that are implantable for use in cancer are especially useful where systemic delivery is limited by toxicity or insufficient tumor exposure. Tumor microenvironment-responsive delivery systems have been widely investigated to achieve selective drug activation and release at tumor sites. Implantable nanoparticle-containing biomaterials can, in principle, exploit such stimuli through localized placement and controlled release mechanisms to achieve sustained drug delivery [48]. Nanoparticle engineering strategies frequently incorporate ligand targeting and peptide functionalization approaches. These strategies improve cellular uptake and therapeutic specificity even if the delivery is local rather than systemic [49,50]. Furthermore, relevant examples demonstrate how nanoparticle formulation parameters affect the performance of cancer drug-delivery nanoparticles and how these parameters may be leveraged in implantable matrices. This concept is illustrated by the enhanced target-specific activity of chitosan-coated, drug-loaded PLGA nanoparticles [51].

In orthopedics, implantable scaffolds and coatings are employed for sustained delivery of anti-inflammatory and antimicrobial agents. The controlled release of a dopamine D1 receptor agonist to augment osteogenic potential is in line with the rationale for implantable local depots in bone regeneration [52] and demonstrates the advantage of sustained presentation of bioactive signals compared with free administration. These findings further highlight the growing importance of coating design. Furthermore, controlled release of ultrasmall silver nanoparticles demonstrates how release of nanoparticles can be harnessed for the treatment of persistent infections linked to implanted materials. This presents an ongoing translational challenge in orthopedic and wound-related implants [53]. Cardiovascular applications continue to be the cornerstone of implantable delivery. There is systematic evidence that design choices affect outcomes when drug-elution is used in acute coronary syndromes [34]. Neurology additionally calls for implantable or localized strategies due to delivery hurdles and the need for sustained dosing. Furthermore, ongoing advances in nanoparticle-based strategies for CNS delivery, as well as next-generation neurotherapeutics, can inform implantable platforms [54,55,56]. In particular, neurotrauma, stroke, and related conditions may greatly benefit from localized delivery.

## 4. Wearable Drug Delivery Devices

Wearable drug delivery systems (WDDs) represent a novel class of external, non-invasive technologies for on-demand drug delivery [57,58]. This approach has been extensively investigated in nanoparticle-based biomaterials engineering, particularly for long-term disease management requiring repeated drug administration [57].

### 4.1. Transdermal and Microneedle-Based Systems

Transdermal microneedle drug delivery systems offer a promising alternative to conventional systemic drug delivery techniques. Microneedles (MNs) have been proposed as a potential strategy to disrupt the barrier function of the stratum corneum to enhance the transport of molecules through the skin. This technique employs micron-sized needles precisely fabricated from diverse biomaterials and geometrical designs to generate temporary aqueous microchannels within the skin architecture [59,60]. Microneedle-based systems have significant advantages in drug delivery because they are non-invasive, self-administered and practically painless, while providing targeted localized drug delivery and avoiding systemic side effects by bypassing hepatic metabolic processing [61,62].

Classification of microneedles depends on their functional properties and the type of therapeutic agent delivered. Collectively, these microneedles range from 50 to 1500 μm in length and 50 to 250 μm in diameter. Solid microneedles are sharp, thin needles that penetrate the stratum corneum barrier to improve drug bioavailability in the dermis. In contrast, coated microneedles are typically coated with water-soluble formulations that dissolve upon contact with interstitial fluid to release the drug. Hollow microneedles are specifically employed for delivery of macromolecular therapeutic agents [61,62,63].

Finally, the “poke-and-release” approach uses dissolving microneedles to enable rapid drug release. Despite these advantages, transdermal and microneedle-based systems can deliver effectively only a limited number of drugs with specific physicochemical characteristics through the skin because the stratum corneum barrier limits drug permeation to therapeutic levels [61].

### 4.2. Nanotechnology-Enabled Wearable Patches

Nanotechnology-enabled wearable patches combine flexible or adhesive device architectures with nanoparticle-containing matrices to support sustained, localized, or on-demand transdermal administration. Nanoparticles may be incorporated into polymer films, hydrogels, adhesives, or reservoir compartments to improve drug stability, enhance interaction with the skin, and regulate delivery over clinically relevant periods [64,65,66,67]. Depending on the intended application, these platforms may also be coupled with biosensors, electrical components, or external controllers to permit responsive or personalized administration. The molecular and physicochemical mechanisms underlying passive and stimuli-responsive drug release are discussed in detail in Section 5.

### 4.3. Integration with Biosensors

The application of biosensors is a new strategy for remote diagnostics and clinical monitoring that offers healthcare practitioners state-of-the-art tools for real-time, longitudinal assessment of patients. Beyond data collection, these systems enable continuous monitoring of physiological signals to promote patient self-management. This technology, when combined with drug delivery systems, is based on two main pillars: high analytical sensitivity and autonomous feedback release. These integrated systems can perform real-time analysis of specific biological markers, thereby providing accurate therapeutic doses, and can be considered a robust solution for the management of chronic conditions such as cancer, diabetes and cardiovascular disease [68].

The integration of biosensors is based on two different designs. Integrated microneedle patches, smart bandages, and wireless contact lenses have demonstrated the use of separate modules for sensing, control, and release [69,70,71]. The alternative incorporates the sensing logic into the biomaterial such that the material directly alters its release behavior in response to local chemical conditions. This material-encoded pathway is exemplified by glucose-responsive microneedle matrices and associated intelligent hydrogel systems, which blur the distinction between biosensor and carrier [72,73].

### 4.4. On-Demand and Patient-Controlled Administration

On-demand and patient-controlled administration represents an important functional advantage of nanoparticle-enabled wearable drug-delivery devices. In these systems, nanoparticle-containing reservoirs, hydrogels, thin films, or microneedle arrays are combined with user-activated or device-controlled components that regulate the timing of administration. Representative examples include mechanically activated microneedles, touch-responsive patches, stretch-triggered elastomeric systems, iontophoretic devices, wireless platforms, and smartphone-controlled delivery systems. These designs allow treatment to be initiated or adjusted according to patient requirements while maintaining the therapeutic payload within a structured device platform [58,74,75,76]. Detailed descriptions of the electrical, magnetic, thermal, pH-responsive, and other stimuli-dependent release mechanisms are provided in Section 5.

Smartphone-controlled microneedle patches combine carrier materials with charged nanovesicles or drug reservoirs and iontophoretic current, allowing precise modulation of drug dosage through adjustment of the electrical intensity and duration [74]. A comparable principle is used in wireless ocular devices, which are miniaturized and require the integration of flexible reservoirs, thin conductive lines, and low-power electronics in a curved contact lens without affecting vision or damaging the eye [71,75]. The smart contact lens device is composed of a biocompatible polymer and contains ultrathin, flexible electrical circuits and a microcontroller chip for real-time electrochemical biosensing, on-demand drug delivery, wireless power management, and data transmission [71].

All these systems are based on a common engineering concept: the best way to give control to the patient is to keep the user-level activation simple and the internal workings of the device highly organized. The patient initiates therapy or sends a wireless command, but the device must control current flow, reservoir access, transport resistance, and dose consistency through this simple interface [58,76]. Representative nanoparticle-enabled implantable and wearable drug delivery platforms, together with their principal applications, advantages, and limitations, are summarized in Table 2.

To complement the qualitative comparison presented in Table 2, selected original studies reporting measurable performance outcomes are summarized in Table 3. The included platforms differ substantially in nanoparticle composition, therapeutic payload, device architecture, experimental model, and analytical endpoints. Therefore, the reported values should be interpreted as representative examples of performance rather than as direct head-to-head comparisons between platforms.

## 5. Controlled and Stimuli-Responsive Drug Release Mechanisms

Controlled and stimuli-responsive drug release mechanisms represent an advanced pharmaceutical approach to the design of nanoparticle-based biomaterials for implantable and wearable biomedical devices. This strategy enables precise spatial and temporal regulation of therapeutic agent delivery in response to specific signals from the device or surrounding tissue microenvironment. The primary objective of these mechanisms is to maintain drug concentrations within the therapeutic window, thereby enhancing therapeutic efficacy and reducing adverse side effects [77].

Controlled release mechanisms can be broadly categorized into passive and stimuli-responsive strategies. Passive systems typically rely on diffusion through a carrier matrix, erosion or degradation of the biomaterial, and osmotic pumping. In contrast, stimuli-responsive platforms introduce an additional level of regulation by coupling drug release to specific biological or externally applied triggers. These stimuli may be endogenous, such as pH gradients, enzymatic activity, or metabolic fluctuations, or exogenous, including temperature changes, electrical and magnetic fields [78,79].

In implantable and wearable devices, integrating stimuli-responsive nanoparticles transforms drug delivery platforms from static depots into responsive therapeutic interfaces. Implantable systems benefit from localized, microenvironment-triggered release that adapts to inflammatory or pathological signals, whereas wearable devices incorporate externally controllable or sensor-integrated mechanisms capable of real-time modulation. Such advancements are particularly relevant in chronic disease management, where therapeutic requirements fluctuate over time [3]. The following sections examine the main classes of stimuli-responsive drug release mechanisms, highlighting their integration within nanotechnology, biomaterials engineering, and bioelectronic medicine.

### 5.1. pH-Responsive Systems

Physiological pH gradients serve as a critical endogenous trigger for drug release in both implantable and wearable devices to achieve site-specific and on-demand release. Diseased tissues such as tumors (pH ~6.5–6.8 extracellularly), chronic wounds (alkaline pH 7.5–8.9), and inflamed microenvironments exhibit deviations from physiological pH (7.4), enabling rational design of acid- or base-labile nanostructures [78,80,81].

pH-responsive nanoparticle-based biomaterials are engineered through three principal molecular design strategies. First, ionizable polymers containing acidic or basic groups undergo protonation or deprotonation in response to the surrounding pH, leading to swelling or solubility changes driven by electrostatic repulsion between polymer chains and enhanced drug diffusion under pathological conditions [78]. Second, drugs can be conjugated to nanocarriers via pH-sensitive covalent bonds (e.g., hydrazone, acetal). These acid-labile linkers are stable at physiological pH but undergo rapid hydrolytic cleavage in acidic environments such as lysosomes or tumor tissue, enabling site-specific release [80]. Third, mesoporous silica nanoparticles may be equipped with nanovalves or polymer caps that block pores at neutral pH and open upon acidification, allowing on-demand release [82]. Together, these strategies provide complementary mechanisms for controlled, pH-triggered drug delivery in implantable and wearable biomedical applications.

In implantable devices, pH-responsive mechanisms are often integrated into coatings or depots for localized delivery. For instance, hydrogel depots have been engineered to release chemotherapeutic agents like doxorubicin or anti-inflammatory drugs for rheumatoid arthritis specifically in acidic pathological sites, reducing systemic toxicity [83]. In wearable and skin-interfaced devices, pH-responsive wound dressings and microneedles are used to treat infected wounds. These “smart” bandages can detect the rise in pH caused by bacterial growth and trigger the release of antibiotics or growth factors like VEGF to accelerate healing [5].

A key limitation of pH-responsive systems is the minimal pH difference between healthy and diseased tissues, which limits selectivity and may lead to premature drug leakage. Tissue buffering and interpatient or site-specific heterogeneity further contribute to unpredictable release kinetics [78,84].

### 5.2. Enzyme-Responsive Systems

Enzyme-responsive systems constitute a highly selective strategy in nanoparticle-based biomaterials for implantable and wearable biomedical devices, as they utilize disease-associated enzymatic dysregulation to achieve activity-triggered drug release. In pathological microenvironments such as tumors, chronic wounds, and inflamed tissues, enzymes including matrix metalloproteinases (MMP-2, MMP-9), hyaluronidase, elastase, lipases, and esterases are overexpressed relative to healthy tissue.

The fundamental design principle involves incorporating enzyme-sensitive chemical bonds or peptide sequences into the biomaterial matrix. These components act as substrates for target enzymes, triggering degradation, conformational changes, or the cleavage of linkers that hold the therapeutic agent. This targeted approach ensures therapeutic agents are released only where and when needed, minimizing systemic exposure and enhancing efficacy [85].

Several classes of enzymes are exploited for device-integrated delivery. Matrix metalloproteinases (MMPs), overexpressed in chronic wounds, inflamed tissues, and tumors, are widely used to trigger release from smart wound dressings and implantable scaffolds for tissue engineering, and MMP-responsive hydrogels have shown success in reversing drug resistance in postoperative glioma treatment [86,87]. Hyaluronidase, overexpressed in the tumor extracellular matrix and secreted by pathogenic microbes, serves as a target for localized cancer therapy and infection control [88]. Lipases and esterases are utilized for on-demand antibiotic release or degradation of polymer shells in specific implant environments [89].

Despite their precision, enzyme-responsive systems face challenges including interpatient variability in enzyme levels, risk of off-target activation, baseline leakage, and the need for kinetic matching between enzymatic cleavage rate and the therapeutic window [85].

### 5.3. Temperature-Responsive Systems

Temperature-responsive systems represent a class of stimuli-responsive biomaterials that utilize localized thermal variations to regulate drug release kinetics in implantable and wearable devices. These systems are commonly engineered using polymers that exhibit a Lower Critical Solution Temperature (LCST), such as poly(N-isopropylacrylamide) (PNIPAM), poly(N-vinyl caprolactam), and thermosensitive poloxamers. Below the LCST, the polymer chains remain hydrated and swollen, whereas above the LCST they undergo a hydrophilic-to-hydrophobic transition, resulting in chain collapse, network contraction, and accelerated drug release [90].

For implantable devices, thermo-responsive hydrogels can be designed as injectable formulations that transition into a stable gel in situ at body temperature, forming a localized drug depot for sustained and targeted drug delivery in applications such as cancer therapy and chronic pain management. Thermo-responsive nanoparticles may also release drugs in response to externally induced hyperthermia, enhancing site-specific delivery while limiting systemic exposure [91]. Recent advances include thermosensor implants based on PNIPAAm-co-PAAm hydrogels, which undergo temperature-dependent volumetric changes for real-time temperature monitoring and controlled drug release [92].

In wearable biomedical devices, temperature-responsive materials are explored for their ability to react to external temperature changes or physiological heat. Hydrogel-based wearables, for example, can be designed for wound healing, where a temperature-responsive dressing might adjust its properties to optimize drug release or protect the wound from contamination [93]. Another example is a thermo-responsive microneedle patch developed for diabetic foot ulcers, in which temperature-sensitive polymers undergo structural changes that modulate drug diffusion and enhance localized release [94]. Despite these advancements, clinical translation remains challenging due to the potential for premature release in naturally hyperthermic inflamed tissues and the need for high-precision temperature regulation [90].

### 5.4. Electrical and Magnetic Stimuli

Electrical and magnetic stimuli-responsive systems enable “on-demand” drug release, providing precise spatiotemporal control that is independent of internal physiological variations. These platforms utilize conductive polymers, electroactive hydrogels, and magnetic nanoparticles to convert applied electrical fields or magnetic stimuli into physicochemical changes that modulate drug release kinetics [84].

Electrical stimuli regulate drug release through iontophoresis, redox-induced bond cleavage, and electro-induced hydrogel deformation. These systems often incorporate conductive nanoparticle fillers, such as carbon nanotubes, graphene, or polypyrrole (PPy), within hydrogel matrices to create efficient electron transport pathways. Wearable examples include magnesium battery-powered patches for psoriasis that use conductive viologen-based hydrogel cathodes to release dexamethasone and tannic acid under low-voltage stimulation [95]. Similarly, iontophoresis-driven microneedle patches enhance transdermal delivery of large molecules like insulin by using electrical repulsion to overcome the stratum corneum barrier [96]. In implantable systems, wirelessly programmed multi-reservoir microchips have achieved controlled release of parathyroid hormone fragments in clinical studies [97].

Magnetic stimuli systems are enabled by magnetic nanoparticles (MNPs) that respond to external magnetic fields, providing non-invasive and deep tissue penetration. In implantable devices, magnetic-responsive scaffolds incorporating magnetic nanoparticles (MNPs) can generate localized hyperthermia under an alternating magnetic field, thereby enhancing protein diffusion and promoting bone regeneration [98]. Targeted drug delivery for conditions like cancer involves guiding MNPs to specific tissues using external magnetic fields, enabling localized drug release and minimizing systemic toxicity [99]. For wearable devices, magnetically triggerable elastomers integrated into 3D printed porous scaffolds offer active, externally controlled drug delivery [100]. Despite the high precision of electrical- and magnetic-stimuli-responsive systems, their clinical translation remains challenging because of the need for complex external hardware and the potential dose-dependent toxicity of magnetic materials [101].

### 5.5. Smart and Self-Regulating Release Platforms

Smart and self-regulating release platforms represent the peak of precision medicine, integrating real-time biosensing with on-demand therapeutic intervention through closed-loop control systems. This ability to respond autonomously to changes in the environment, without external human intervention, is crucial for maintaining precise drug concentration, enhancing efficacy, and minimizing adverse effects over extended periods. These platforms are generally categorized into self-sustained (autonomous) and externally triggered systems, both of which utilize nanoparticle-based biomaterials to deliver treatments tailored to a patient’s immediate physiological needs [102,103].

Self-sustained systems operate without external intervention by utilizing bio-responsive materials that undergo structural transformations, such as swelling, dissociation, or degradation, in response to internal biological markers. Glucose-responsive insulin delivery represents a prototypical example of closed-loop therapy. Systems incorporating glucose oxidase (GOx) function as biochemical transducers, catalyzing the conversion of glucose to gluconic acid, thereby reducing local pH and triggering insulin release from pH-sensitive nanocarriers [104].

Externally triggered closed-loop systems integrate bioelectronics to achieve precise spatiotemporal control of drug delivery. These devices, often in the form of smart wearable patches, utilize sensors to monitor physical or biochemical parameters (e.g., sweat glucose, tremors, or skin temperature). The detected signal is transmitted to a control unit, which activates an actuator to initiate drug release. For example, a wearable patch for diabetes management can detect hyperglycemia in sweat and activate an integrated heater to melt phase-change material (PCM) coatings on microneedles, thereby releasing metformin [105]. Similarly, wearable systems for Parkinson’s disease can detect muscle tremors and trigger drug release from thermoresponsive mesoporous silica nanoparticles [102,106].

The evolution of these “intelligent” platforms is further accelerated by advancements in 3D printing, which enable the fabrication of patient-specific devices with high resolution, and wireless communication (NFC/Bluetooth), which allows for remote monitoring and adjustment by healthcare providers. While these systems offer transformative potential for managing chronic conditions like epilepsy, cardiovascular diseases, and autoimmune disorders, their clinical translation requires addressing challenges in long-term biocompatibility, reproducible scalability, and the accuracy of real-time sensing [3,102,103]. The main stimuli-responsive drug release mechanisms and their applications in implantable and wearable biomedical devices are summarized in Table 4.

**Figure 4:**
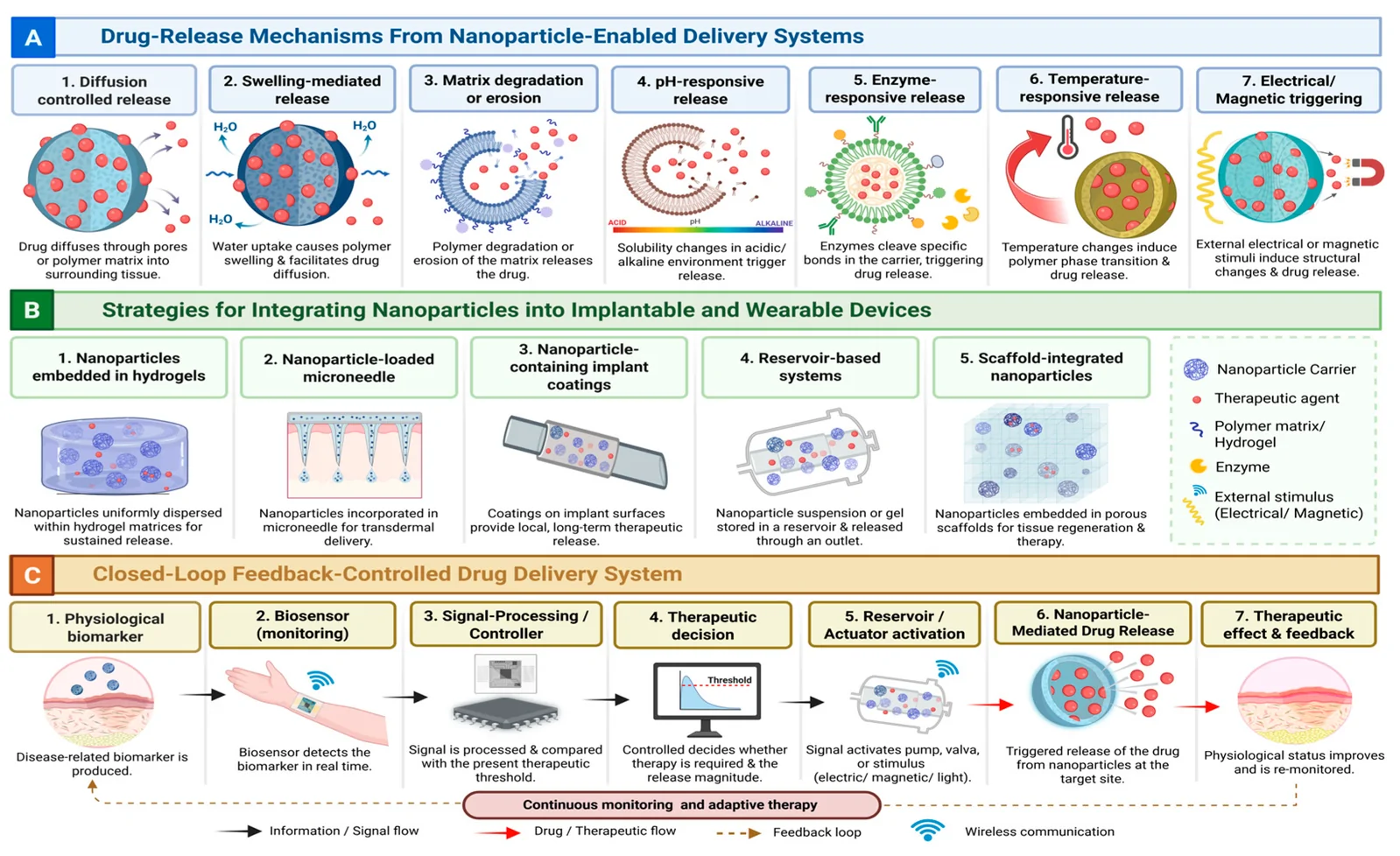
Schematic overview of nanoparticle-enabled drug-release mechanisms, device integration strategies, and closed-loop feedback-controlled delivery. (**A**) Major drug-release mechanisms from nanoparticle-enabled delivery systems, including diffusion-controlled release, swelling-mediated release, matrix degradation or erosion, pH-responsive release, enzyme-responsive release, temperature-responsive release, and electrically or magnetically triggered release. (**B**) Representative strategies for integrating nanoparticles into implantable and wearable devices, including hydrogel-embedded nanoparticles, nanoparticle-loaded microneedles, nanoparticle-containing implant coatings, reservoir-based systems, and scaffold-integrated nanoparticles. (**C**) General workflow of a closed-loop feedback-controlled drug-delivery system, in which physiological biomarkers are continuously monitored by a biosensor, processed by a control unit, and translated into therapeutic decisions that activate the reservoir or actuator, trigger nanoparticle-mediated drug release, and subsequently generate therapeutic feedback for adaptive treatment. Created with BioRender.com.

The principal mechanisms governing drug release from nanoparticle-enabled devices, the major strategies used to integrate nanocarriers into implantable and wearable platforms, and the operational sequence of closed-loop feedback-controlled delivery are summarized schematically in Figure 4. The figure highlights the transition from passive diffusion- and degradation-mediated release to externally triggered and biosensor-guided systems capable of adaptive therapeutic delivery [77,78,79,80,81,82,83,84,85,86,87,88,89,90,91,92,93,94,95,96,97,98,99,100,101,102,103,104,105].

## 6. Surface Functionalization and Biointerface Engineering

Surface functionalization and biointerface engineering are essential for improving the biological performance of nanoparticle-based biomaterials utilized in implantable and wearable drug delivery systems. The interactions between nanoparticles and the biological environment significantly affect biodistribution, cellular uptake, immune recognition, and the device’s long-term performance. In numerous biomedical contexts, unmodified nanoparticles quickly interact with plasma proteins and immune cells, forming a biomolecular corona that can significantly change the nanoparticles’ behavior [107]. Therefore, rational surface-engineering strategies are needed to improve targeting efficiency, minimize immune clearance, and enhance biocompatibility in nanoparticle-based drug-delivery systems [108,109,110].

One of the most common methods in nanoparticle surface engineering is the addition of targeting ligands that facilitate selective binding to certain cells or tissues. Targeting ligands, including antibodies, peptides, aptamers, small molecules such as folate, and proteins such as transferrin, can be conjugated to nanoparticle surfaces to enhance receptor-mediated uptake and improve therapeutic accuracy. Early studies in nanomedicine indicated that ligand-functionalized nanoparticles could significantly increase drug accumulation at disease sites compared with non-targeted systems [111,112]. Recent advancements have expanded these techniques through advanced bioconjugation strategies that allow precise ligand orientation and density on nanoparticle surfaces, thereby optimizing receptor binding and internalization [109,110,113]. In the context of implantable drug-delivery systems, ligand-functionalized nanoparticles can improve targeted therapeutic delivery to diseased tissues such as tumors, inflamed areas, or damaged organs.

Another key aspect of biointerface engineering is creating antifouling and antimicrobial coatings to prevent unwanted protein adsorption and microbial growth. Biofouling is considered a major challenge for both implantable and wearable devices. This is because proteins can build up on the surfaces of biomaterials, trigger inflammatory responses, and impair device performance. Polyethylene glycol (PEG) coatings have traditionally been used to minimize protein adsorption and prolong nanoparticle circulation time [114]. More recently, alternative antifouling materials such as zwitterionic polymers, hydrophilic brushes, and biomimetic surface coatings have been developed to further reduce nonspecific interactions with biological components [115,116]. Along with antifouling properties, antimicrobial coatings containing silver or copper nanoparticles or antimicrobial peptides have been investigated to lower infection risks related to implantable medical devices [117,118]. These methods are especially important for long-term biomedical implants, in which microbial contamination and biofilm formation might threaten the safety and performance of the device.

In addition to reducing biofouling, advanced surface engineering techniques also aim to improve tissue integration and immune response modulation. The interaction between biomaterials and the host immune system is a major determinant of device performance and longevity. Implantable biomaterials often provoke a foreign-body reaction characterized by macrophage recruitment, chronic inflammation, and fibrotic encapsulation. Recent studies have shown that the surface features of biomaterials, such as chemistry, roughness, and hydrophilicity, can influence immune cell behavior and macrophage polarization [119,120]. For example, certain surface chemistries can encourage anti-inflammatory macrophage phenotypes, promoting tissue regeneration rather than fibrosis. Additionally, biomaterials engineered with immunomodulatory molecules or bioactive peptides have been shown to regulate inflammatory responses and enhance integration with nearby tissues [121]. These immune-instructive materials are increasingly studied for implantable drug delivery systems and regenerative medicine, especially for their ability to promote recovery and minimize rejection in patients with implants.

Surface engineering is also crucial for maintaining the long-term stability and functionality of nanoparticle-based biomedical devices. For implantable systems, maintaining structural integrity and biological compatibility over extended periods is crucial for consistent therapeutic performance. Surface modifications can improve nanoparticle stability, prevent aggregation, and protect therapeutic payloads from premature degradation. Moreover, designing a robust biointerface helps maintain device functionality in complex physiological environments where proteins, enzymes, and mechanical stresses might affect device performance [122]. Ultimately, these strategies help ensure that the therapeutic effects of the drug delivery system are achieved without compromising patient safety. In wearable drug delivery systems, surface engineering strategies are also used to enhance interactions between nanoparticles and biological tissues, such as the skin, thus promoting effective drug transport and reducing irritation.

Overall, progress in surface functionalization and biointerface engineering has significantly improved the effectiveness of nanoparticle-based drug delivery systems for implantable and wearable biomedical devices. By incorporating targeting ligands, antifouling coatings, antimicrobial surfaces, and immune-modulating materials, researchers can create multifunctional nanoparticle systems capable of targeted therapeutic delivery while maintaining long-term biocompatibility. Ongoing progress in biomaterial surface engineering will likely facilitate the creation of next-generation smart biomedical devices with enhanced therapeutic effectiveness, increased patient safety, and greater prospects for successful clinical use.

## 7. Key Challenges and Limitations

### 7.1. Material-Specific Toxicity, Biodistribution, and Accumulation

The toxicity, biodistribution, clearance, and long-term accumulation of nanoparticle-based biomaterials vary substantially according to material composition, particle size, surface charge, biodegradability, and route of exposure. Polymeric nanoparticles composed of biodegradable materials such as PLGA, PLA, PCL, chitosan, and related polymers are generally designed to undergo hydrolytic or enzymatic degradation. Their safety therefore depends not only on the parent polymer but also on the rate of degradation, local accumulation of degradation products, residual solvents, and the possibility of prolonged inflammatory responses when degradation is incomplete or poorly matched to tissue-healing timelines [14,15,16,17,18,28,29,30].

Lipid-based nanoparticles, including liposomes, solid lipid nanoparticles, and nanostructured lipid carriers, are generally considered more biodegradable because their components resemble physiological lipids. Nevertheless, their biological behavior is influenced by lipid composition, oxidation, PEGylation, protein adsorption, and uptake by the mononuclear phagocyte system. Repeated exposure or prolonged retention may promote accumulation in the liver and spleen, complement activation, or formulation-specific immune reactions, particularly when surface properties alter clearance pathways [19,20,21,28,29].

Inorganic nanoparticles present greater concerns regarding long-term persistence because many are not readily biodegradable. Gold, silica, and iron oxide nanoparticles may exhibit prolonged tissue retention, organ accumulation, oxidative stress, or dose-dependent cytotoxicity depending on particle size, coating, surface chemistry, and administered dose. Iron oxide nanoparticles may enter physiological iron-processing pathways after degradation, whereas gold nanoparticles may persist for extended periods. Mesoporous silica and calcium phosphate systems may be more degradable under appropriate physiological conditions, but their dissolution rate and degradation products remain highly dependent on composition and porosity [22,23,24,28,29,30].

Hybrid and composite nanoparticles combine multiple material classes and therefore may inherit the safety concerns of each individual component. Their multicomponent structure can complicate prediction of degradation, clearance, and toxicological behavior, particularly when persistent inorganic cores are combined with biodegradable polymeric or lipid shells. Accordingly, safety evaluation should consider the intact hybrid particle, each constituent material, degradation products, and potential interactions among components rather than assuming that the biocompatibility of one component applies to the complete system [24,25,26,27,28,29,30].

For implantable platforms, localized administration may reduce systemic exposure but can increase long-term tissue contact, local particle retention, inflammatory reactions, and regional accumulation. Wearable systems generally produce lower systemic exposure, although repeated transdermal administration may still result in cumulative uptake. Material-specific evaluation should therefore include local and systemic biodistribution, organ retention, degradation kinetics, clearance pathways, repeated-dose toxicity, and long-term histopathological assessment.

### 7.2. Long-Term Stability and Degradation

Long-term stability and degradation behavior differ substantially among polymeric, lipid-based, inorganic, and hybrid nanoparticle systems. These differences influence structural integrity, drug retention, release kinetics, shelf life, and performance after incorporation into implantable or wearable devices. In all systems, changes in temperature, pH, ionic strength, enzymatic activity, and interactions with proteins and other biological components may promote aggregation, surface reorganization, premature degradation, or loss of therapeutic activity [109,123].

Polymeric nanoparticles undergo degradation primarily through hydrolytic or enzymatic cleavage of the polymer matrix. Their degradation rate depends on polymer composition, molecular weight, crystallinity, hydrophilicity, particle morphology, and local physiological conditions. For example, PLGA degradation can be adjusted through the lactic acid-to-glycolic acid ratio and polymer molecular weight, whereas more slowly degrading polymers such as PCL may provide prolonged structural stability. However, uneven degradation, autocatalytic effects, or local accumulation of acidic degradation products may alter drug-release kinetics and produce premature or incomplete release [14,15,16,17,18,109].

Lipid-based nanoparticles are generally more susceptible to physical and chemical instability during storage. Liposomes may undergo phospholipid oxidation, hydrolysis, membrane fusion, aggregation, and leakage of encapsulated drugs. Solid lipid nanoparticles may experience lipid crystallization and polymorphic transitions that promote drug expulsion, whereas nanostructured lipid carriers generally reduce this problem through the incorporation of liquid lipids, although oxidation and phase separation may still occur [19,20,21]. Consequently, lipid composition, antioxidant protection, storage temperature, and packaging conditions are particularly important for maintaining the stability of lipid-based systems.

Inorganic nanoparticles generally exhibit greater structural stability than biodegradable organic nanocarriers but often degrade slowly or remain persistent under physiological conditions. Gold nanoparticles may retain their core structure for extended periods, while mesoporous silica and calcium phosphate nanoparticles can undergo gradual dissolution depending on particle porosity, surface modification, and local pH. Iron oxide nanoparticles may degrade through intracellular processing and enter physiological iron pathways. Although this structural persistence may support prolonged device performance, it can also complicate clearance and increase the risk of long-term tissue retention [22,23,24,28].

Hybrid and composite nanoparticles display more complex stability profiles because their individual components may degrade at different rates. A biodegradable polymeric or lipid shell may degrade before an inorganic core, potentially exposing the core and altering particle interactions with tissues. Similarly, weak interfacial compatibility between components may promote phase separation, aggregation, drug leakage, or changes in release behavior. Their stability must therefore be evaluated at the level of both the individual components and the complete integrated system [24,25,26,27].

These material-specific differences are especially important when nanoparticles are incorporated into implantable or wearable devices. Implantable platforms must maintain predictable degradation and release behavior over extended periods in a continuously changing biological environment, whereas wearable systems must additionally tolerate storage, repeated mechanical deformation, temperature variation, and exposure to moisture. Therefore, long-term evaluation should include particle size, surface charge, morphology, drug content, degradation products, release kinetics, mechanical integrity, and biological performance under both storage and clinically relevant conditions. Conventional short-term laboratory assays may not adequately predict long-term in vivo behavior, highlighting the need for accelerated stability studies and extended physiological testing [124,125].

### 7.3. Immune Response and Foreign Body Reaction

On entering the body, nanoparticles rapidly adsorb proteins from the surrounding fluids, forming a protein corona that reshapes how the immune system perceives them; as a result, chemically identical materials can behave very differently in vivo. Persistence depends heavily on the route of administration, with some formulations cleared quickly and others retained in tissues for prolonged periods. Immune recognition is governed largely by the composition of the corona, whereas surface properties such as size, charge, and chemistry dictate cellular uptake, with macrophages proving especially efficient at engulfing nanoparticles. Materials that appear inert in vitro may therefore provoke pronounced immune responses once implanted.

Early stability and apparent immune tolerance do not guarantee durable compatibility. Nanodevices that remain in situ frequently trigger successive waves of inflammation, which, rather than resolving, may persist long after implantation. This sustained response can lay down concentric layers of connective tissue that encapsulate and isolate the device, altering drug diffusion so that release is slowed or accelerated in ways that gradually erode efficacy rather than causing abrupt failure [109,126]. The immune system is, moreover, exquisitely sensitive to subtle material differences: minor variations in synthesis, composition, or surface chemistry can yield substantially different biological outcomes, making formulation optimization difficult and batch-to-batch consistency hard to preserve [109,124]. Because biological responses cannot be reliably inferred from material composition alone, immune and foreign body reactions remain principal barriers to clinical translation. Current strategies aim not to abolish immune activity but to blunt its harmful components while preserving therapeutic efficacy. However, achieving dependable immune modulation over long periods remains unresolved and continues to raise legitimate concerns about the long-term safety and clinical acceptance of these therapies.

### 7.4. Scale-Up and Manufacturing Challenges

Notwithstanding substantial progress in laboratory-scale nanoparticle design, large-scale manufacturing continues to pose formidable obstacles. Most nanoparticle systems demand tightly controlled conditions to preserve uniform size, surface characteristics, and drug loading, and reproducing those conditions at production scale is difficult, since even minor changes in equipment configuration can markedly alter particle behavior [109,124]. Small deviations in temperature, mixing profile, or reaction time may cause particles to aggregate rather than form uniformly, producing uneven drug loading and inconsistent release rates that ultimately compromise therapeutic performance.

Because nanoparticles possess an exceptionally high surface-area-to-volume ratio, even minor imperfections carry greater consequences than in bulk materials, and manufacturing variances that would be tolerable in conventional pharmaceuticals can disrupt the delicate biological interactions on which nanomedicines depend. The problem intensifies when nanoparticles are incorporated into higher-order dosage forms such as implants or patches, where every production step adds technical demands and further opportunities for variability. Ensuring batch-to-batch consistency is therefore difficult, and it stands as a significant obstacle to regulatory approval.

From an industrial standpoint, scale-up must reconcile cost efficiency, throughput, and quality control. Techniques such as microfluidic synthesis and multilayer surface modification perform well in the laboratory but are frequently too costly to scale [125,127], so that otherwise promising candidates may remain confined to early-stage development for want of practical manufacturing solutions. Bridging the gap between nanoscale precision and industrial feasibility thus remains a critical challenge: successful translation demands manufacturing methods that are reliable, scalable, compliant with regulatory requirements, and economically viable.

The economic feasibility of nano-enabled implantable and wearable drug-delivery systems depends on more than the cost of nanoparticle synthesis alone. Total production costs also include raw-material qualification, aseptic processing or sterilization, specialized analytical characterization, integration of the nanocarrier with the device, packaging, storage, and long-term quality-control testing. Multifunctional systems that incorporate targeting ligands, responsive materials, biosensors, microelectronics, or wireless components may provide greater therapeutic control but also require more complex manufacturing processes and supply chains, thereby increasing development and unit costs [125,127]. These expenses may ultimately affect reimbursement, affordability, and access, particularly when the platform offers only a modest clinical advantage over an established conventional therapy. Conversely, higher initial manufacturing costs may be economically justified when the device reduces dosing frequency, hospitalization, systemic toxicity, treatment-related complications, or the need for repeated clinical visits. Therefore, economic evaluation should consider the complete product lifecycle and compare the additional manufacturing and implementation costs with measurable improvements in clinical outcomes, patient adherence, healthcare-resource utilization, and device longevity [125,127].

## 8. Translational and Regulatory Perspectives

**Figure 5:**
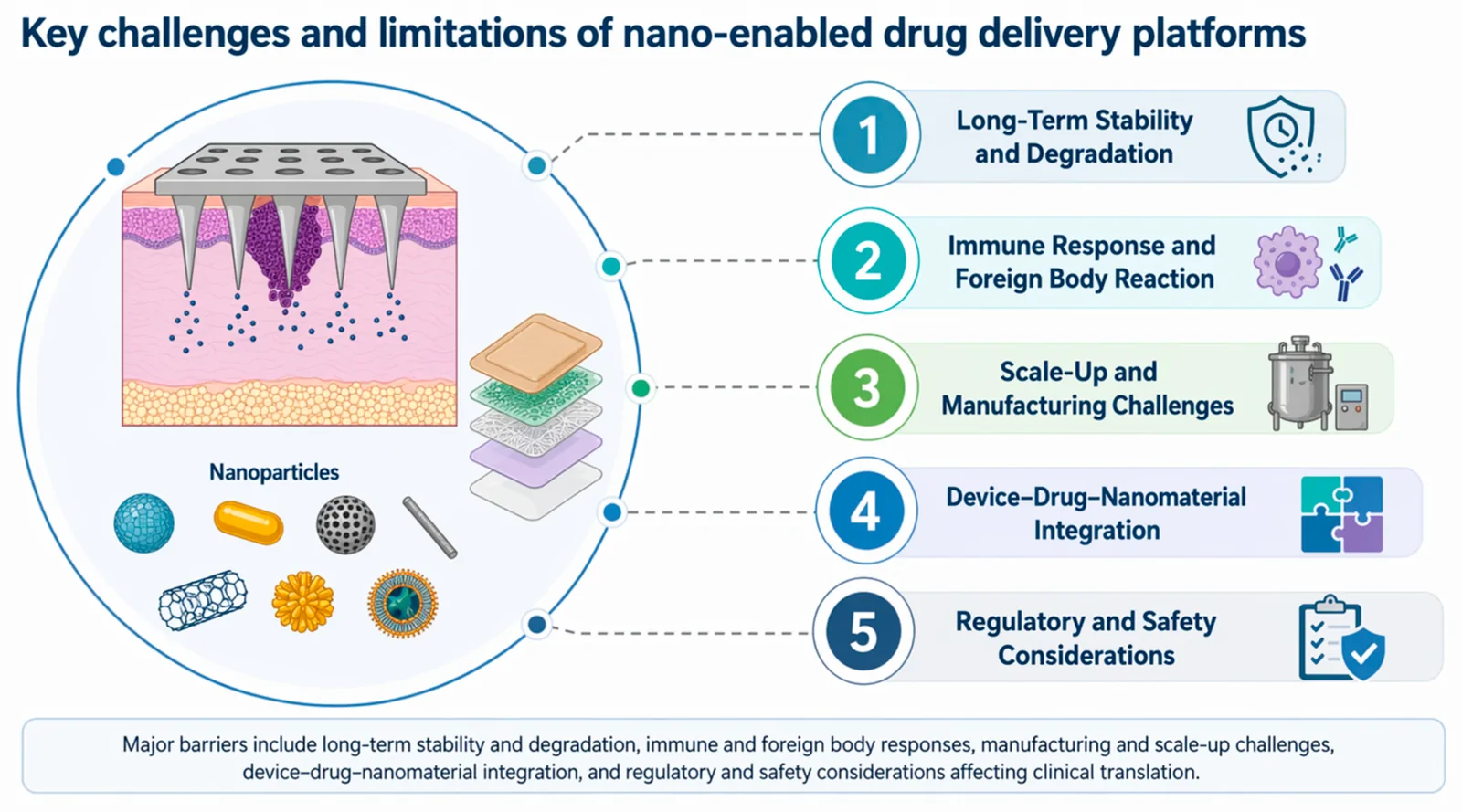
Key challenges and limitations of nano-enabled drug delivery platforms. Major barriers include long-term stability and degradation, immune and foreign body responses, manufacturing and scale-up challenges, the integration of device, drug, and nanomaterial components, and regulatory and safety considerations affecting clinical translation.

The clinical translation of nanoparticle-based biomaterials integrated into implantable and wearable drug delivery devices requires overcoming a complex set of scientific, engineering, manufacturing, and regulatory challenges. Unlike conventional pharmaceutical products, these platforms are frequently classified as combination products that integrate nanomaterials, medical devices, and therapeutic agents, thereby necessitating multidisciplinary evaluation strategies. Successful translation depends not only on demonstrating safety and efficacy but also on ensuring manufacturing reproducibility, long-term stability, regulatory compliance, and post-market safety monitoring. As illustrated in Figure 5, the translational pathway encompasses sequential stages from formulation design and preclinical assessment to clinical implementation and regulatory approval, with distinct considerations for implantable and wearable systems.

### 8.1. Preclinical Evaluation Strategies

**Figure 6:**
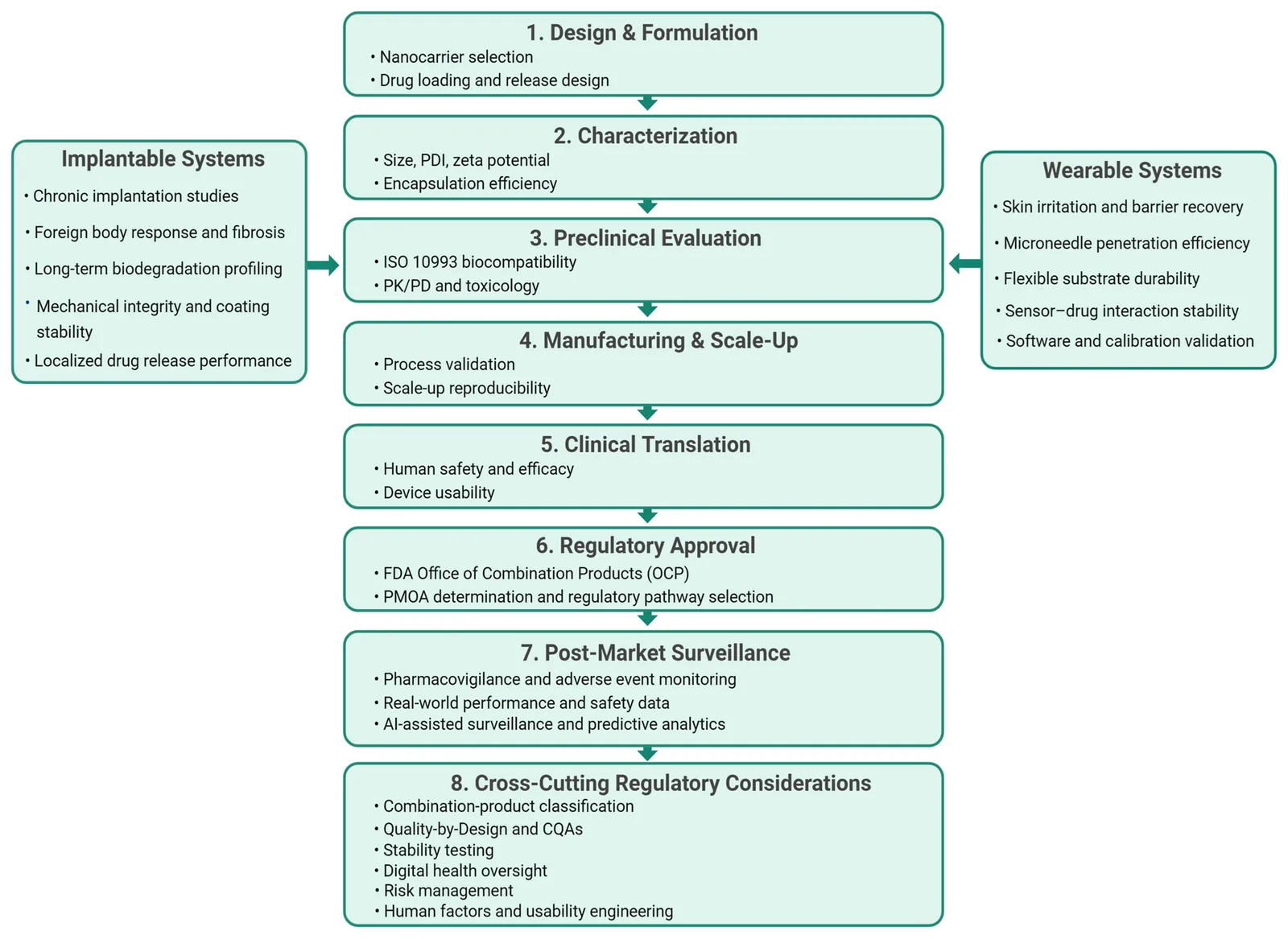
Translational and regulatory roadmap for nano-enabled implantable and wearable drug delivery systems. The figure summarizes the progression from nanoparticle formulation and characterization to preclinical testing, manufacturing, clinical translation, regulatory approval, and post-market surveillance, highlighting the distinct translational challenges and regulatory requirements associated with implantable and wearable drug delivery platforms. Created with BioRender.com.

The successful translation of nanoparticle-based biomaterials integrated into implantable and wearable drug delivery devices requires a multidimensional framework encompassing formulation design, preclinical evaluation, manufacturing, clinical translation, regulatory approval, and post-market surveillance. As illustrated in Figure 6, the translational pathway involves distinct scientific, engineering, and regulatory considerations for implantable and wearable platforms, which collectively determine their successful clinical implementation [127,128,129].

According to ISO 10993 standards, biocompatibility evaluations must be performed to assess cytotoxicity, sensitization, genotoxicity, hemocompatibility, and chronic implantation [130]. For implantable nano-enabled systems, long-term foreign body reactions (FBR), in vivo fibrotic encapsulation, and nanoparticle degradation byproducts must be comprehensively evaluated [119,131]. Chronic inflammation and macrophage polarization are particularly relevant in polymeric and lipid-based nanoformulations [132].

When evaluating pharmacokinetics (PK) and pharmacodynamics (PD), researchers must account for localized drug reservoirs and prolonged nanoparticle-mediated drug release. This often deviates from traditional systemic pharmacokinetic distribution models [133]. For orthopedic [134], cardiovascular, and neural implants, large animal models are recommended to more accurately mimic physiological biomechanical loading and tissue remodeling dynamics.

For wearable nano-enabled platforms such as microneedle arrays and transdermal patches, preclinical studies should evaluate penetration efficiency, barrier recovery, dermal immune responses, and dose uniformity under physiological motion conditions [135,136]. Increasingly, computational modeling and in silico simulations of release kinetics and device–nanoparticle interactions are being employed prior to first-in-human studies [137].

### 8.2. Clinical Translation Hurdles

Although strong preclinical data exist, translation into clinical practice remains limited. A major obstacle is the reproducibility of manufacturing processes. The synthesis conditions of nanoparticles strongly influence their physicochemical properties, including particle size, size distribution, surface charge, morphology, and drug-loading efficiency. These variations complicate process standardization, batch-to-batch reproducibility, and large-scale manufacturing [138,139]. Variability from one batch to another may significantly alter release kinetics and safety profiles. These translational inconsistencies frequently result in regulatory delays or requests for additional comparability data.

Another challenge is sterilization. The use of gamma irradiation, autoclaving, or exposure to ethylene oxide may affect the integrity of polymers, lipid bilayers, or even the active pharmaceutical ingredient [140]. Thus, the design of nanoformulations that are compatible with various sterilization techniques is necessary for the purposes of regulatory submissions.

Storage can also present long-term stability issues. Lipid-based nanoparticles may undergo aggregation or drug leakage, while polymeric systems may undergo hydrolytic degradation [141]. Shelf-life requirements and storage conditions are established through stability testing in accordance with ICH guidelines [142].

Clinical trial design is complicated by multiple factors. For instance, combination products necessitate the concurrent validation of device reliability, nanoparticle stability, and pharmacological activity [143]. Moreover, devices that have electrically or magnetically triggered release systems pose additional complexities to endpoint determination and risk assessment [144].

### 8.3. Clinical Lessons from Approved and Unsuccessful Nanoformulations

Clinically approved nanoformulations provide important lessons for the translation of nanoparticle-enabled implantable and wearable drug-delivery systems. Doxil^®^/Caelyx^®^, a PEGylated liposomal formulation of doxorubicin, demonstrated that nanocarrier encapsulation can preserve antitumor efficacy while substantially reducing cardiotoxicity compared with conventional doxorubicin, although formulation-specific adverse effects such as palmar–plantar erythrodysesthesia remain relevant [145]. Abraxane^®^, a solvent-free albumin-bound nanoparticle formulation of paclitaxel, achieved improved response rates and eliminated the need for toxic solvent-based excipients and routine premedication, illustrating the clinical value of reformulating a poorly soluble drug using a biocompatible carrier [146]. Similarly, the NAPOLI-1 trial showed that liposomal irinotecan combined with fluorouracil and folinic acid improved survival in patients with metastatic pancreatic cancer following gemcitabine-based therapy, supporting the importance of altered pharmacokinetics and prolonged intratumoral drug exposure [147]. Nevertheless, not all clinically evaluated nanoformulations have been successful. In the Phase III HEAT trial, lyso-thermosensitive liposomal doxorubicin combined with radiofrequency ablation did not significantly improve progression-free or overall survival in the overall study population [148]. This outcome illustrates how the clinical performance of externally triggered nanocarriers may depend on precise control of device-related parameters, treatment duration, patient selection, and consistency between the nanocarrier mechanism and the clinical protocol. More broadly, successful translation appears to require a clearly demonstrated therapeutic advantage over existing treatments, reproducible manufacturing, predictable pharmacokinetics, manageable toxicity, and an appropriately designed clinical trial, whereas many failures have resulted from insufficient efficacy rather than unacceptable toxicity [149].

### 8.4. Regulatory Pathways for Combination Products

Nanotechnology-enabled wearable and implantable systems for drug delivery integrate biological components with device parts and in some cases with digital health technologies, and therefore typically fall under the definition of ‘combination products’. Regulatory classification primarily depends on the product’s primary mode of action (PMOA), which describes the principal contributor to the therapeutic effect of the product, whether it is the drug, the device, or a biological entity [150]. Jurisdictional assignment in the United States falls to the FDA Office of Combination Products (OCP). The OCP provides a full description of the product to assess mechanism of action and assigns it to the appropriate division, whether that be the Center for Drug Evaluation and Research (CDER), Center for Devices and Radiological Health (CDRH), or Center for Biologics Evaluation and Research (CBER) [151]. Depending on the risk classification, novelty, and intended use, the regulatory pathway may require a New Drug Application (NDA) submission if the pharmacological component is predominant, a Premarket Approval (PMA) for high-risk, device-led systems, or 510(k) clearance if the device is deemed substantially equivalent to an already legally marketed device [152]. While the risk-based approach offers a structured means for navigating the complexities of device-led systems, it appears to fall short when applied to systems that utilize nanoscale technologies and materials, where interdependent drug release kinetics, performance of the device, and biodegradation of device materials are concerns that require simultaneous evaluation of safety, effectiveness, and quality across multiple regulatory domains.

In the European Union, the Medical Device Regulation (EU MDR 2017/745) requires some systems to undergo rigorous clinical assessment and comply with post-market surveillance requirements [153]. If medicinal products are involved, consultation with the competent authorities is required.

Global regulatory harmonization remains limited, and nanotechnology-specific guidance on the standardization of characterization requirements is still evolving [154]. In regard to AI-enabled wearable systems, guidelines on Software as a Medical Device (SaMD) issued by the International Medical Device Regulators Forum (IMDRF) are of significance [155].

Wearable systems incorporating a biosensor and an adaptive dosing algorithm that automatically adjusts the dose are likely to be characterized by emerging regulatory frameworks that overlap with respect to devices, drugs, and digital health [156]. The absence of nano-specific harmonized regulatory frameworks continues to represent a major global translational barrier. Beyond technical and regulatory requirements, commercialization is influenced by the cost and complexity of nanoparticle production, device fabrication, sterilization, packaging, quality-control testing, and long-term stability assessment. These burdens may be particularly important for multicomponent systems that combine a drug, nanocarrier, device, sensor, or digital control element, because each component must be manufactured consistently and validated both independently and as part of the integrated product [143,153,154,155]. Limited access to specialized manufacturing infrastructure and the need to demonstrate clear clinical benefit over established dosage forms may further slow market adoption. Accordingly, successful translation requires early integration of scalable manufacturing and cost considerations into product development rather than addressing these issues only after preclinical validation [153,154,155].

### 8.5. Standardization and Quality Control

Standardization is central to the regulatory acceptance and successful clinical translation of nano-enabled implantable and wearable drug delivery systems. Comprehensive characterization must encompass both physicochemical and functional performance parameters. Critical attributes include particle size distribution, polydispersity index (PDI), zeta potential, drug loading, encapsulation efficiency, release kinetics, and long-term colloidal stability. Analytical techniques such as dynamic light scattering (DLS), electron microscopy (TEM and SEM), and spectroscopic methods are routinely employed to ensure consistency, reproducibility, and product quality. In addition, in vitro release studies should be conducted under physiologically relevant conditions to establish meaningful correlations between laboratory performance and anticipated in vivo behavior [157].

In addition to physicochemical characterization, manufacturing processes for nano-enabled implantable and wearable drug delivery systems must comply with Good Manufacturing Practice (GMP) and Quality System Regulations (QSR) to ensure consistency, traceability, and regulatory acceptability [158]. Given the complexity of nanoparticle-based formulations, Critical Quality Attributes (CQAs), including particle size distribution, polydispersity index (PDI), surface charge, drug loading efficiency, encapsulation efficiency, and in vitro release profiles, should be defined early in development and rigorously validated during scale-up, as these parameters directly influence therapeutic performance [159]. For implantable systems, additional engineering validation is required to confirm mechanical integrity under physiological stress conditions and to establish predictable degradation kinetics that correlate with sustained drug release over the intended therapeutic period. In wearable systems, quality requirements extend to device durability, calibration stability of integrated biosensors, and, where applicable, verification and validation of software components governing adaptive dosing algorithms in accordance with emerging Software as a Medical Device (SaMD) frameworks [160].

Post-market surveillance programs should incorporate structured real-world performance data collection, adverse event reporting, and long-term safety monitoring, particularly for non-biodegradable nanomaterials. The major regulatory, manufacturing, and quality control considerations relevant to nano-enabled combination products are summarized in Table 5.

## 9. Future Perspectives

The next generation of nanoparticle-based biomaterials for implantable and wearable drug delivery is expected to move beyond static controlled-release platforms towards intelligent therapeutic systems capable of sensing, decision-making, and dose adaptation [161,162]. This transition reflects the convergence of nanotechnology, bioelectronics, artificial intelligence (AI), and precision medicine, in which biomaterials, sensors, electronics, and computational tools operate as an integrated therapeutic ecosystem rather than as isolated components [163,164]. However, the clinical translation of emerging nano-enabled and nanoelectronic drug-delivery technologies remains uneven, with substantial differences in technological maturity and clinical integration [165]. The following perspective is therefore organized by translational readiness, distinguishing near-term technologies that are already entering practice from mid-term and longer-horizon concepts that remain largely preclinical. In the near term, one of the most advanced directions is adaptive and increasingly automated drug delivery. Wearable and implantable technologies are increasingly being integrated with intelligent therapeutic platforms to support responsive and on-demand dosing with minimal patient intervention [166,167,168]. Systems of this type are no longer purely conceptual; glucose-sensing insulin-delivery loops provide an established clinical precedent for closed-loop therapeutic control [166]. Digital design and advanced manufacturing represent another important direction. Machine-learning approaches have already been applied to predict the 3D-printing performance of large numbers of drug-delivery formulations, illustrating how computational methods can accelerate formulation and manufacturing optimization [169]. Nanotechnology is also increasingly being incorporated into orthopedic drug-delivery systems and implants, where material selection, biocompatibility, and localized therapeutic delivery remain important translational considerations [170]. At a broader systems level, digital-twin approaches are emerging in healthcare as computational frameworks for integrating patient-specific data and supporting increasingly individualized therapeutic decision-making [171]. In parallel, advances in 3D-printed wearable sensors demonstrate the growing convergence of additive manufacturing, sensing technologies, and personalized healthcare platforms [172]. Smart and stimuli-responsive biomaterials further extend these concepts by enabling materials to alter their properties or function in response to environmental conditions [173]. Four-dimensional printing provides a more specific example: dual-responsive chitosan microneedles have been designed to change their geometry after insertion, demonstrating how stimulus-responsive structures can improve mechanical anchoring and functional performance [174]. Reports of 5D and higher-dimensional printed tissue scaffolds extend these concepts toward increasingly complex control of scaffold geometry and function [175]; however, these technologies remain considerably less mature than established 3D-printing approaches. Over the mid-term, biomimetic nanoparticle strategies are particularly promising. Nanoparticles coated with natural cell membranes are being investigated to reproduce selected biological surface properties and improve interactions with biological systems. Red-blood-cell-membrane-coated nanoparticles demonstrate the functional potential of erythrocyte-derived coatings [176], while platelet-membrane-coated nanoparticles have been investigated as biomimetic platforms for targeted therapy [177]. Such approaches illustrate the potential of cell-membrane coatings to improve biological interactions and targeted nanoparticle delivery. Further from clinical realization are nanorobotic and highly autonomous therapeutic systems. Nanorobotic platforms capable of precisely controlled modes of motion illustrate the potential for nanoscale devices to navigate complex environments and support future targeted therapeutic applications [178]. Nevertheless, substantial challenges related to control, safety, reproducibility, and integration with clinically practical delivery systems remain before such approaches can become routine. Another important direction is the development of self-powered and battery-free biomedical devices. Battery-free implantable systems have demonstrated the feasibility of sustained physiological monitoring without conventional implanted batteries [179], while triboelectric and piezoelectric nanogenerators are being actively investigated as energy-harvesting technologies for self-powered healthcare monitoring devices [180]. These approaches may help reduce dependence on conventional power sources and support longer-term operation of future wearable and implantable therapeutic platforms. Across all of these directions, the decisive barrier is unlikely to be the discovery of new materials alone. As established in Section 6 and Section 7, the principal obstacles include reproducible manufacturing at the Good Manufacturing Practice (GMP) scale and the absence of harmonized, nanotechnology-specific regulatory frameworks for drug-device combination products. Consequently, progress in the coming years may depend less on simply expanding the repertoire of nanocarriers and more on standardizing their characterization, demonstrating batch-to-batch consistency, and defining the regulatory pathways through which such systems can reach patients. The technologies described above will mature at very different rates, and realistic near-term impact is most likely where nanoparticle-based biomaterials are paired with device architectures and manufacturing routes that are already close to clinical acceptance. Emerging research is increasingly moving beyond passive controlled-release systems toward adaptive, programmable, and digitally integrated therapeutic platforms. Artificial intelligence and machine-learning approaches may support formulation optimization, dose prediction, device calibration, and individualized treatment adjustment. Integration with digital therapeutics and real-time biosensing could further enable closed-loop systems that continuously interpret physiological signals and modify drug release accordingly. Self-powered wearable and implantable devices based on triboelectric, piezoelectric, or other energy-harvesting mechanisms may reduce dependence on conventional batteries, while biomimetic nanoparticle coatings and increasingly sophisticated responsive biomaterials may improve biological interaction, targeting, and device functionality. Collectively, these developments suggest a transition toward multifunctional systems that combine sensing, computation, responsive materials, and precisely controlled therapeutic delivery [44,102,103,104,105,161,162,163,164,165,166,167,168,169,170,171,172,173,174,175,176,177,178,179,180].

## 10. Conclusions

Nanoparticle-based biomaterials have emerged as a powerful platform for advancing controlled drug delivery in implantable and wearable biomedical devices. Polymeric, lipid-based, inorganic, and hybrid nanocarriers each offer distinct physicochemical properties that govern drug loading, release kinetics, and tissue compatibility, and their integration within device architectures enables localized, sustained, and stimuli-responsive therapy while limiting systemic toxicity. When combined with biosensing and bioelectronic components, these systems begin to function as responsive therapeutic interfaces capable of adjusting drug release in response to physiological signals, with drug-eluting implants, smart wound dressings, and microneedle-based platforms illustrating the clinical relevance of the approach for chronic and site-specific conditions.

Despite this progress, translation to routine clinical use remains constrained. The central difficulties are not conceptual but practical: maintaining long-term stability, controlling degradation, managing the foreign body response, ensuring sterilization compatibility, achieving reproducible manufacturing at scale, and navigating the regulatory classification of drug–device–nanomaterial combination products. These challenges are interdependent, and addressing them will require characterization standards and quality-control frameworks that are currently only partially defined for nanoscale systems.

Over the next 5–10 years, the systems most likely to achieve clinical implementation are biodegradable polymeric and lipid-based nanocarriers integrated into relatively simple and established device formats, particularly microneedle patches, nano-enabled wound dressings, drug-eluting implant coatings, and localized biodegradable depots. These platforms offer a more favorable balance between therapeutic benefit, biocompatibility, manufacturing feasibility, and regulatory readiness. More complex biosensor-controlled and externally triggered systems may also advance, but their widespread adoption will depend on reliable device integration, long-term safety, scalable manufacturing, and clear clinical superiority over existing therapies.

## Figures and Tables

**Table 1:** Comparison of major nanoparticle-based biomaterials for implantable and wearable drug delivery.

Nanoparticle Class	Representative Systems	Key Structural and Physicochemical Features	Drug-Loading and Release Characteristics	Major Advantages	Main Considerations or Limitations	Potential Relevance to Implantable and Wearable Devices	References
**Polymer-based nanoparticles**	PLGA, PLA, PCL, PEG-based nanoparticles; chitosan, alginate, gelatin, hyaluronic acid, silk fibroin; polymeric micelles	Tunable size, surface chemistry, molecular weight, copolymer composition, and degradation profile. Natural polymers may provide mucoadhesion or intrinsic biological activity.	Can sustain release from days to months. Polymeric micelles encapsulate hydrophilic and hydrophobic agents through core–shell architectures. Release can be adjusted through polymer composition and degradation.	High formulation versatility; programmable degradation; amenable to ligand functionalization, PEGylation, and stimuli-responsive modification.	Performance depends on polymer composition, degradation rate, surface properties, and interactions with biological fluids. Degradation products and long-term biocompatibility require evaluation.	Implantable depots, biodegradable matrices, hydrogels, microneedles, and systems requiring sustained or stimuli-responsive delivery.	[14,15,16,17,18]
**Lipid-based nanoparticles**	Liposomes, solid lipid nanoparticles (SLNs), nanostructured lipid carriers (NLCs), and lipid-polymer hybrid systems	Structural similarity to biological membranes. Liposomes contain phospholipid bilayers around an aqueous core; SLNs contain solid lipid matrices; NLCs combine solid and liquid lipids.	Liposomes can co-encapsulate hydrophilic drugs in the aqueous core and lipophilic drugs in the bilayer. SLNs and NLCs support controlled release and loading of poorly soluble drugs. Some systems enable temperature- or pH-triggered release.	Favorable biocompatibility; flexible drug loading; potential use of approved lipid excipients; adaptable surface modification; scalable formulation options.	Lipid organization, storage stability, drug expulsion, protein adsorption, and foreign-body interactions must be controlled.	Wearable patches, implantable reservoirs, localized delivery systems, and responsive drug-release platforms.	[19,20,21]
**Inorganic nanoparticles**	Gold nanoparticles, mesoporous silica nanoparticles, iron oxide nanoparticles/SPIONs, and calcium phosphate nanoparticles	Distinct optical, magnetic, porous, or mineral-like properties. Gold nanoparticles exhibit surface plasmon resonance; mesoporous silica has high surface area and tunable pores; SPIONs respond to magnetic fields; calcium phosphate resembles bone mineral.	Mesoporous structures permit high loading and pore-gated release. Gold nanoparticles support near-infrared-triggered release. Iron oxide nanoparticles enable magnetic guidance, hyperthermia, and imaging. Calcium phosphate combines delivery with osteoconductivity.	External-stimulus responsiveness; precise surface conjugation; high loading capacity in porous systems; potential theranostic functionality; relevance to bone applications.	Many inorganic systems are not readily biodegradable. Long-term persistence, organ accumulation, dose-dependent toxicity, and clearance require careful assessment.	Remotely triggered implants, image-guided systems, magnetically responsive platforms, orthopedic implants, and specialized on-demand delivery devices.	[22,23,24,28,29,30]
**Hybrid and composite nanoparticles**	Lipid-polymer hybrid nanoparticles; SPION-containing lipid or polymer systems; nanoparticle-loaded hydrogels, fibers, ceramics, and scaffold composites	Combine two or more material classes to integrate structural stability, biocompatibility, controlled release, magnetic responsiveness, or scaffold functionality.	Polymeric cores can provide sustained release while lipid shells improve biological interactions. Multiple nanoparticle types may support co-delivery with independently controlled release profiles.	Multifunctionality; combined therapeutic and diagnostic capability; improved encapsulation; potential for multistage or multidrug delivery; adaptable mechanical and biological properties.	Greater compositional and manufacturing complexity; more demanding characterization, reproducibility, degradation assessment, and regulatory evaluation.	Multifunctional implants, nano-enabled scaffolds, remotely controlled devices, regenerative platforms, and advanced wearable systems.	[24,25,26,27]

**Table 2:** Representative implantable and wearable nanoparticle-enabled drug delivery platforms.

Platform Category	Representative System	Nanoparticle or Biomaterial	Release Approach	Main Application	Key Advantage	Main Limitation	References
**Implantable**	Drug-eluting stents	Nanoparticle-containing coatings and polymer matrices	Sustained localized release	Cardiovascular disease	Reduced restenosis and localized drug exposure	Foreign-body response and coating stability	[31,34,35,36,37]
**Implantable**	Nano-enabled scaffolds	Lipid nanoparticles, SLNs, calcium phosphate, chitosan, and hydrogels	Matrix-controlled and degradation-mediated release	Bone repair and tissue regeneration	Structural support combined with sustained drug delivery	Mechanical and release-profile optimization	[38,39,40,41]
**Implantable**	Hydrogel depots	Nanoparticle-loaded hydrogels and extracellular-vesicle systems	Diffusion, swelling, and matrix degradation	Cancer, pain, and osteoarthritis	Long-term localized therapy	Variable degradation and burst release	[42,43,44,45,46,47]
**Implantable**	Tumor-responsive depots	Functionalized PLGA and chitosan-coated nanoparticles	Localized and microenvironment-responsive release	Cancer therapy	Improved target specificity and reduced systemic exposure	Tumor heterogeneity and limited penetration	[48,49,50,51]
**Implantable**	Orthopedic antimicrobial systems	Silver nanoparticles and drug-releasing coatings	Controlled local release	Implant-associated infection	Sustained antimicrobial activity	Potential nanoparticle toxicity	[52,53]
**Wearable**	Transdermal patches	Polymeric, lipid-based, and stimuli-responsive nanoparticles	Passive diffusion and stimulus-triggered release	Chronic disease management	Non-invasive and sustained administration	Skin-barrier limitations	[57,64,65,66,67]
**Wearable**	Microneedle systems	Dissolving, coated, hollow, or nanoparticle-integrated microneedles	Rapid, sustained, or iontophoretic delivery	Insulin, macromolecules, and localized therapy	Minimally invasive and patient-friendly	Limited drug loading and skin variability	[59,60,61,62,63,69,74]
**Wearable**	Biosensor-integrated patches	Smart hydrogels, microneedles, and sensor-controlled reservoirs	Closed-loop or feedback-controlled release	Diabetes and chronic disease monitoring	Real-time sensing and autonomous dosing	Device complexity and calibration requirements	[68,69,70,71,72,73]
**Wearable**	Smart contact lenses	Flexible reservoirs, electrical circuits, and microcontrollers	Wireless and electrically controlled release	Ocular therapy and biosensing	Continuous monitoring with on-demand delivery	Miniaturization and ocular safety	[71,75]
**Wearable**	Patient-controlled systems	Iontophoretic patches and smartphone-controlled devices	Electrically activated release	Personalized dosing	Direct patient control	Dependence on electronics and power supply	[58,74,75,76]

**Table 3:** Representative quantitative performance and translational status of selected nanoparticle-enabled implantable and wearable drug-delivery platforms.

Platform	Nanoparticle/Biomaterial and Payload	Key Quantitative Performance	Release/Delivery Profile	Application and Model	Development Stage	Reference
**Smartphone-powered iontophoresis-microneedle array patch**	Insulin-loaded positively charged nanovesicles	FITC-insulin cumulative permeation reached 25.48 ± 1.14 µg after 5 h at 3 mA; approximately 2.24-fold higher than microneedles without iontophoresis; penetration depth was approximately 280 µm.	Electrically adjustable, on-demand transdermal delivery; normoglycemia maintained for approximately 6.8 h.	Type 1 diabetes; in vitro rabbit-skin permeation and diabetic rat model.	Preclinical	[74]
**Fully integrated mesoporous microneedle-iontophoresis closed-loop system**	Mesoporous microneedles with insulin solution	Free diffusion released 5.8 ± 0.15% of insulin over 180 min; 0.5 mA iontophoresis enhanced delivery by approximately 2.3-fold. Normoglycemia lasted 3.3 h in diabetic rats.	Basal diffusion combined with electrically triggered bolus delivery in a sensor-coupled closed-loop system.	Diabetes monitoring and treatment; diabetic rat model.	Preclinical	[69]
**Wireless smart contact lens with flexible drug-delivery system**	Electrically triggered genistein reservoir integrated into a smart contact lens	Electrical activation released 89.97 ± 37.10% of the loaded genistein in PBS.	Wireless, pulsatile, on-demand ocular drug release.	Diabetic retinopathy-related therapy and glucose monitoring; diabetic rabbit model.	Preclinical	[71]
**Glucose-responsive dual-hormone microneedle patch**	Polymeric microneedles containing insulin and a glucagon analogue	Minipig patches contained 4.8 mg insulin and 1.6 mg glucagon analogue; glucose regulation was maintained for more than 24 h in minipigs.	Glucose-dependent, bidirectional closed-loop release of insulin and glucagon analogue.	Type 1 diabetes; diabetic mouse and minipig models.	Preclinical large-animal validation	[72]

**Table 4:** Stimuli-responsive drug release mechanisms in implantable and wearable devices.

Stimulus or System	Representative Responsive Materials	Release Mechanism	Representative Device Applications	Main Advantages	Main Limitations	References
pH-responsive	Ionizable polymers, acid-labile linkers, and gated mesoporous silica nanoparticles	Protonation or deprotonation induces swelling or solubility changes; acid-labile bonds cleave; pore caps open under altered pH	Implantable **hydrogel depots, tumor-targeted systems, smart wound dressings, and microneedles**	Site-specific and microenvironment-triggered release with reduced systemic exposure	Small pH differences between healthy and diseased tissues, buffering effects, heterogeneity, and premature leakage	[78,80,81,82,83,84]
Enzyme-responsive	Enzyme-cleavable polymers, peptide linkers, hydrogels, and nanoparticle shells responsive to MMPs, hyaluronidase, lipases, or esterases	Disease-associated enzymes cleave linkers or degrade the carrier matrix, releasing the therapeutic payload	Smart wound dressings, implantable scaffolds, localized cancer systems, and infection-responsive devices	High biochemical selectivity and localized activation at diseased sites	Interpatient variability, off-target activation, baseline leakage, and the need to match cleavage kinetics to the therapeutic window	[85,86,87,88,89]
Temperature-responsive	PNIPAM, poly(N-vinyl caprolactam), poloxamers, thermosensitive hydrogels, and thermo-responsive nanoparticles	Temperature changes across the LCST cause polymer dehydration, collapse, contraction, or altered diffusion	Injectable in situ-forming depots, implantable thermosensors, wound dressings, and microneedle patches	Tunable thermal activation and compatibility with externally induced hyperthermia	Premature activation in inflamed tissues and the need for precise temperature control	[90,91,92,93,94]
Electrical stimuli	Conductive polymers, electroactive hydrogels, carbon nanotubes, graphene, polypyrrole, and iontophoretic systems	Iontophoresis, redox-triggered bond cleavage, or electrically induced hydrogel deformation regulates release	Battery-powered wearable patches, iontophoretic microneedles, and programmable implantable microchips	Rapid, externally controllable, and precise on-demand dosing	Dependence on power sources and electronics, device complexity, and potential tissue or material safety concerns	[84,95,96,97]
Magnetic stimuli	Magnetic nanoparticles, SPION-containing scaffolds, and magnetically responsive elastomers	External magnetic fields guide particles, generate localized hyperthermia, or mechanically alter the carrier to enhance release	Magnetically activated implants, bone-regeneration scaffolds, targeted cancer systems, and wearable porous scaffolds	Non-invasive remote activation and relatively deep tissue penetration	Need for external hardware, dose-dependent toxicity, heating control, and long-term persistence of magnetic materials	[98,99,100,101]
Smart and self-regulating platforms	Glucose oxidase-responsive nanocarriers, biosensor-coupled microneedles, phase-change coatings, smart patches, and bioelectronic control units	Real-time sensing is coupled to autonomous or externally triggered activation of the drug reservoir in a closed-loop system	Glucose-responsive insulin delivery, sweat-sensing diabetes patches, Parkinson’s disease wearables, and remotely monitored systems	Adaptive dosing, real-time feedback, improved personalization, and reduced need for manual intervention	Sensor accuracy, calibration, communication reliability, long-term biocompatibility, scalability, and system integration	[102,103,104,105]

**Table 5:** Translational and regulatory considerations for nano-enabled implantable and wearable drug delivery systems.

Translational Aspect	Implantable Drug Delivery Systems	Wearable Drug Delivery Systems	Major Translational Challenges	Regulatory Frameworks and Standards	Sources
**Preclinical evaluation**	Chronic implantation studies, foreign body response (FBR), biodegradation kinetics, and long-term tissue compatibility assessment	Skin irritation, penetration efficiency, barrier recovery, repeated-use safety, and dermal immunogenicity assessment	Limited predictive value of animal models and lack of standardized nano-specific testing platforms	ISO 10993 biocompatibility standards and nanomaterial safety assessment frameworks	[130,131,132,135,136,154]
**Manufacturing and scale-up**	Sterilization-sensitive nanoformulations, implant mechanical integrity, and reproducible nanoparticle incorporation	Flexible substrate integration, microfabrication reproducibility, and sensor–drug component assembly	Batch-to-batch variability, manufacturing cost, and scalability constraints	Good Manufacturing Practice (GMP) requirements, Quality System Regulations (QSR), and scale-up guidance	[138,139,140,156,158]
**Stability and storage**	Long-term degradation kinetics, drug-release consistency, and structural integrity during implantation	Environmental stability, sensor–drug interactions, and mechanical durability during repeated use	Difficulty predicting long-term performance under physiological and environmental stress conditions	International Council for Harmonisation (ICH) stability guidelines	[141,142,156]
**Regulatory classification**	Predominantly drug-led combination products; NDA, BLA, or PMA pathways depending on the primary mode of action (PMOA)	Device-, software-, or combination-product classification, with potential digital health integration	Ambiguity in product classification and regulatory jurisdiction	FDA Office of Combination Products and PMOA framework; EU Medical Device Regulation 2017/745	[150,151,152,153,154,155]
**Quality control and standardization**	Critical quality attributes, including particle size, PDI, encapsulation efficiency, and release kinetics	Device performance, calibration stability, software validation, and sensor accuracy	Lack of harmonized standards for nano-enabled combination products	Quality-by-Design and critical quality attribute principles; Software as a Medical Device guidance	[155,157,158,159,160]
**Post-market surveillance**	Long-term implant surveillance, adverse-event reporting, and biodegradation monitoring	Real-world performance monitoring, remote data collection, and AI-assisted safety surveillance	Long-term safety assessment and integration of digital health data	EU MDR post-market surveillance requirements and digital health regulatory oversight frameworks	[153,155,156]

## Data Availability

No new data were created or analyzed in this study. Data sharing is not applicable to this article.
